# Multi-Gene Phylogenetic Approach for Identification and Diversity Analysis of *Bipolaris maydis* and *Curvularia lunata* Isolates Causing Foliar Blight of *Zea mays*

**DOI:** 10.3390/jof8080802

**Published:** 2022-07-29

**Authors:** Nazia Manzar, Abhijeet Shankar Kashyap, Avantika Maurya, Mahendra Vikram Singh Rajawat, Pawan Kumar Sharma, Alok Kumar Srivastava, Manish Roy, Anil Kumar Saxena, Harsh Vardhan Singh

**Affiliations:** 1 Plant Pathology Lab, ICAR-National Bureau of Agriculturally Important Microorganisms, Maunathbhanjan 275103, India; rajawat.mvs@gmail.com (M.V.S.R.); pawan112000@gmail.com (P.K.S.); 2 Molecular Biology Lab, ICAR-National Bureau of Agriculturally Important Microorganisms, Maunathbhanjan 275103, India; 3 Division of Plant Pathology, ICAR-Indian Agricultural Research Institute, New Delhi 110012, India; avantika.maurya@gmail.com; 4 Microbial Technology Unit I, ICAR-National Bureau of Agriculturally Important Microorganisms, Maunathbhanjan 275103, India; aloksrivastva@gmail.com; 5 ICAR-National Bureau of Agriculturally Important Microorganisms, Maunathbhanjan 275103, India; manish.nbaimj@gmail.com (M.R.); saxena461@yahoo.com (A.K.S.); drharsh2006@rediffmail.com (H.V.S.)

**Keywords:** *Bipolaris maydis*, *Curvularia lunata*, ITS, GAPDH, LSU, maize, multi-gene phylogeny

## Abstract

*Bipolaris* species are known to be important plant pathogens that commonly cause leaf spot, root rot, and seedling blight in a wide range of hosts worldwide. In 2017, complex symptomatic cases of maydis leaf blight (caused by *Bipolaris maydis*) and maize leaf spot (caused by *Curvularia lunata*) have become increasingly significant in the main maize-growing regions of India. A total of 186 samples of maydis leaf blight and 129 maize leaf spot samples were collected, in 2017, from 20 sampling sites in the main maize-growing regions of India to explore the diversity and identity of this pathogenic causal agent. A total of 77 *Bipolaris maydis* isolates and 74 *Curvularia lunata* isolates were screened based on morphological and molecular characterization and phylogenetic analysis based on ribosomal markers—nuclear ribosomal DNA (rDNA) internal transcribed spacer (ITS) region, 28S nuclear ribosomal large subunit rRNA gene (LSU), D1/D2 domain of large-subunit (LSU) ribosomal DNA (rDNA), and protein-coding gene-glyceraldehyde-3-phosphate dehydrogenase (GAPDH). Due to a dearth of molecular data from ex-type cultures, the use of few gene regions for species resolution, and overlapping morphological features, species recognition in *Bipolaris* has proven difficult. The present study used the multi-gene phylogenetic approach for proper identification and diversity of geographically distributed *B*. *maydis* and *C*. *lunata* isolates in Indian settings and provides useful insight into and explanation of its quantitative findings.

## 1. Introduction

Maize (*Zea mays* L.) is one of the most diverse crops with great adaptability under a range of broad agro-climatic zones. It is the third-most commonly cultivated cereal after wheat and rice, is known as the ‘Queen of the Cereals’ globally because of its significant potential for genetic yield among cereals, and is grown in approximately 150 m ha across about 160 countries with wide soil properties, climate, biodiversity, and management practices [1]. Maize is cultivated on 9.2 million hectares in India, with an average yield of 2965 kg/ha and a total production of 27.8 million tons [2,3]. The major producing states of maize in India are, by zone, East: Bihar, Orissa; North: Uttar Pradesh, Punjab, Haryana, Himachal Pradesh, Jammu, Kashmir; West: Rajasthan, Gujarat, Maharashtra; South: Andhra Pradesh; Central: Madhya Pradesh [4,5].

On a global basis, maize is affected by 112 diseases, but in Indian agroclimatic conditions, there are 35 main diseases that attack maize [6]. Worldwide yield losses in maize up to 9% have been estimated due to fungal, bacterial, and nematode diseases [7]. The severe maize diseases are spread across a broad disease spectrum and include downy mildew, rust, leaf blight, and stalk rot [8]. These diseases also include maydis leaf blight (MLB), one of the most devastating diseases that has emerged as an economically significant problem [9].

Worldwide, *Curvularia* and *Bipolaris* species attack cereal and grasses, causing leaf spot diseases on rice, maize, sorghum, wheat, and barley, resulting in severe grain yield losses [10,11,12,13]. An important genus of plant pathogens is *Bipolaris* (an anamorph of the ascomycetes genus *Cochliobolus*), with more than 100 species. *Bipolaris* species mostly attacks members of the poaecae family and causes root rot, leaf spot or blight, seedling blight, and ear rot and blight [14]. Due to being seed-borne in nature, *Bipolaris* spp. causes significant damage to field crops, mostly as post-harvest damage that ultimately impairs market value [15,16]. *Bipolaris* spp. has caused severe epidemics, such as the Great Bengal famine, Northern leaf spot blight, and Southern corn leaf blight, which ultimately caused significant economic losses in recent decades [17].

Worldwide, in maize producing regions, the maydis leaf blight, also known as Southern corn leaf blight (SCLB), is a severe maize crop disease caused by *Bipolaris maydis*, an anamorph of *Cochliobolus heterostrophus*. It is considered as a graminicolous species of *Helminthosporium* [11,17,18]. In USA and UK, *Bipolaris maydis* caused severe epidemics in 1970. The decline in the development of maize is possibly due to maydis leaf blight in India.

Maydis leaf blight (MLB or SCLB) generally occurs in moderately humid and warm conditions with temperatures of 20–32 °C and causes potential damage and yield losses in major maize-growing regions of India. During the Kharif season, the disease spreads rapidly throughout India and is most prevalent in plains, highlands, and peninsular regions [19]. Sharma and Rai (2000) reported that maydis leaf blight caused by *B. maydis* qualifies as a major disease of maize is capable of inflicting significant losses in productivity, to the extent of 41 percent [20].

*Bipolaris* and *Curvularia* species mainly have been characterized based on cultural and morphological characteristics in the past few decades. Dothideomycetes fungi, the most diverse class of fungi, include *Curvularia* and *Bipolaris* species [21]. All members of *Drechslera* and *Exserohilum* once grouped to the graminicolous *Helminthosporium* species [12]. Species of *Bipolaris* were previously known as *Helminthosporium*. The genus *Helminthosporium* is divided into four genera based on taxonomic refinements: *Drechslera*, *Bipolaris*, *Exserohilum*, and *Curvularia*. They are morphologically similar, and particularly, these four genera are called helminthosporioid fungi [12,18]. Some of these related genera have caused maize leaf spot and mixed infections, caused by the genera *Curvularia* and *Exserohilum*, *C*. *intermedia*, *C*. *lunata*, *C*. *eragrostidis*, *C*. *pallescens*, *C*. *clavata*, *C*. *spicifera*, *C. papendorfii* and *C*. *inaequalis* [22,23], while leaves, sheaths, and maize bracts were reportedly infected by *E*. *rostratum* and *Exserohilum turcicum*, which have also been reported to cause extreme leaf spot in maize [24,25].

Under field conditions, the symptoms of maydis leaf blight caused by those species were prevalent and complex. It is difficult to identify these species reliably based on symptoms alone. Despite their significance, species identification remains problematic in *Bipolaris* and *Curvularia* since numerous species have morphological traits that overlap, making it difficult to differentiate between these two genera under different conditions [12,26]. It might be difficult to distinguish between the genera and species of *Bipolaris* and *Curvularia*. Due to substantial intraspecies and interspecies variability and the difficulty of generating sexual phases under laboratory circumstances, asexual morphological traits are insufficient to correctly identify species [26,27]. However, combined study can differentiate these two genera based on sexual, conidia morphology, and molecular studies.

*Curvularia* has conidia with overly enlarged intermediate cells, which contribute to the curvature, whereas *Bipolaris* has a continuous curvature throughout the conidium duration. In addition, conidia are usually longer in *Bipolaris* than those in *Curvularia* [28]. Based on the morphology of ascospores, *Curvularia* forms loosely packed ascospores in the ascus, while *Bipolaris* produces supercoiled ascospores [29].

Representatives of the Consortium for the Barcode of Life’s Fungal Working Group (FWG) evaluated different markers, three nuclear ribosomal DNA regions (ITS, LSU, SSU), and one protein-coding gene (RPB1), and proposed the ITS region as the key fungal barcode marker [30]. Species identification [31], species level phylogenies [32], and DNA barcoding studies [33] have all been conducted in the region. ITS data in the International Nucleotide Sequence Database (INSD: GenBank, EMBL, and DDBJ) demonstrated that this region is not equally variable in all fungi taxa, although most studies have designated it as the standard barcode marker for fungi.

These taxa have minimal or no barcode gaps in the ITS sequence, according to Stielow et al., 2015 [34]. The ITS region alone may not be sufficient for some species identification. Mycologists, particularly those working with ascomycetous fungi, frequently explore the feasibility of a two-marker barcoding system for fungi [7]). LSU [35,36] and protein-coding genes can be employed with ITS [36]. Using slowly evolving protein-coding genes, in particular, can significantly improve the phylogenetic resolution of deep divergences [37].

*Bipolaris* species are now typically identified based on morphological traits; however, other species exhibit numerous similarities, and conidial characteristics are frequently varied depending on isolates and culture circumstances. Due to ambiguities in morphological characteristics, DNA sequences of multiple loci are preferred to molecular identification and define *Bipolaris* and *Curvularia* species [28,38]. Currently, phylogenetic analysis studies are based on protein-coding gene-glyceraldehyde-3-phosphate dehydrogenase (GAPDH). Ribosomal markers of rDNA internal transcribed spacer (ITS) and a large subunit of nuclear ribosomal DNA (LSU) have been used to evaluate the phylogenetic relationships among the *Bipolaris* genus isolates and with their sister genera [11,28,38]. The ITS, LSU regions, and GAPDH gene phylogeny provided a better resolution for taxa at the terminal clades, which has also been reported for other Dothideomycetes [39,40,41]. Based on these loci, phylogenetic studies have allowed *Cochliobolus* (sexual morph) species to be reallocated to either *Bipolaris* or *Curvularia* [29].

RAxML (Randomized Axelerated Maximum Likelihood) is a tool for inferring complex phylogenetic trees using maximum likelihood in a sequential and parallel way. It can also be used to perform post-analysis on phylogenetic tree sets, alignment analyses, and short-read evolutionary placement. RAxML is among the most widely used and effective software methods for creating a maximum likelihood (ML) tree. This would be the most accurate method for evaluation, but it is also the most resource-intensive. The model developed by RAxML is believed to be a good evolution model. Under this model, there was no major change in the likelihood of the inferred tree fitting the genuine tree [42].

It is difficult to distinguish between species solely based on their morphological characteristics because many of their morphological features overlap. Bhunjan et al., 2020 estimated 132 species epithets in Index Fungorum under *Bipolaris*, of which 15 had been moved to *Curvularia*, and only 43 are approved as *Bipolaris* species [43].

Based on the above studies, the combined study of morphological and molecular data gave a precise and correct method for identifying *Bipolaris* spp. Thus, the objectives of this study were as follows: (i) identification of *Bipolaris maydis* as the cause of maydis leaf blight, and *Curvularia lunata* as the cause of leaf spot of maize based on morphological analysis, (ii) precise identification and phylogenetic analysis based on ITS, GAPDH, LSU, and D1 and D2 regions of LSU to know the diversity of *Bipolaris maydis* and *Curvularia lunata* isolates in different geographical locations of major maize-growing regions in India.

## 2. Materials and Methods

### 2.1. Collection of Maydis-Leaf-Blight-Symptomatic Samples from Major Maize-Growing Regions of India

#### Sampling

During 2017, leaf samples showing characteristic symptoms of maydis leaf blight and leaf spot were collected from Bihar, Uttar Pradesh, Punjab, and Uttarakhand, covering five agro-climatic zones as follows: Western Himalayan Region, Uttarakhand; Lower Gangetic Plains region, Eastern Bihar; Middle Gangetic Plains region, parts of Uttar Pradesh and Bihar; Upper Gangetic Plains region, central and western parts of Uttar Pradesh and the Hardwar and Udham Nagar districts of Uttarakhand; Trans Gangetic Plains region, Punjab, under maize-cropping regions in India (Table 1). Around 186 symptomatic samples of maydis leaf blight were collected based on the characteristic disease symptoms of diamond-shaped lesions that vary in size, with 3–22 mm length and 2–6 mm width, that become rectangular- or spindle-shaped lesions with chlorotic halos at later stages. In the case of leaf spot of maize, 129 maize-leaf-spot-symptomatictic samples in the early stages of maize leaves that had yellow necrotic spots, which eventually expand to circular, spindle-shaped, oval, or strip lesions, were collected. Twenty sampling districts were covered during sampling, and their district locations are depicted in Supplementary Figure S1.

### 2.2. Isolation and Morphological Characterization of Pathogens

The symptomatic tissue was cut from the margins of lesions and surface-sterilized with 1% sodium hypochlorite for 1 min and then rinsed with distilled water three times. The surface-sterilized leaf tissues (5–6 mm^2^) with lesions were placed on Petri plates containing potato dextrose medium (HiMedia, Mumbai, India) amended with chloramphenicol (0.05 g/L) (HiMedia, Mumbai, India) and incubated at 28 ± 2 °C for three days [44]. The agar disc (7 mm) containing the mycelia from the 3-day-old colonies was placed on the center of a Petri plate with fresh PDA and then incubated at 28 ± 2 °C. The pure cultures were obtained through single-spore isolation [29] and maintained on agar slants with regular subculturing.

Observations based on cultural characteristics of the different isolates were recorded over a 15-day incubation period of PDA containing sporulating cultures. Three replications of each isolate were maintained. Microscopic features of isolates, i.e., conidia shape, size, number of septa, color were taken as parameters by using a Olympus BX41 Epi and Trans Fluorescence Microscope (Olympus, Tokyo, Japan) for differentiation of genera [45]. While measuring the morphological features of different genera, thirty observations were randomly documented. The isolates were used to study the morphological characteristics described earlier [10,12,45,46].

Conidia and conidiophores were mounted in distilled water and viewed in an Olympus BX41 Phase Contrast and Dark Field Microscope (Olympus, Tokyo, Japan). Measurements of conidial width were recorded from the widest part of each conidium. Olympus BX41TF Epi and Trans Fluorescence Microscope (Olympus, Hamburg, Germany) measured their lengths and widths based on previous studies [10,12]. On average, 30 measurements of conidial length and width of each isolate was recorded. Mean ± standard deviation for length and width ranges of each conidium was recorded.

*Bipolaris maydis* isolates were cultured on PDA (Himedia, Mumbai, India) for 15 days at 28 °C. Actively growing fungal cultures of agar blocks were cut and fixed overnight at 0.1 M sodium phosphate buffer (pH 7.3) containing 2% glutaraldehyde. In phosphate buffer, each fungal mat was thoroughly washed three times for 15 min and samples were dehydrated for 15 min by using a series of graded ethanol (10, 20, 30, 40, 50, 60, 70, 80, 90, and 100%).The dehydrated and fixed samples of *Bipolaris maydis* isolates were further dried for 5 min with CO_2_ and then immediately fixed on aluminum stubs and sputter-coated with carbon in a Polaron E-500 sputter coater (Polaron Equipment, Watford, England) and viewed under a visible scanning electron microscope (S3400N-Hitachi, Tokyo, Japan) immediately.

Koch’s postulates test [47] for pathogenicity of the isolates of *Bipolaris maydis* and *Curvularia lunata* were carried out in the glass house. In glass house, five maize seeds of Kanchan variety were sown in 45 cm plastic pots containing sterilized soil and were kept in glass house at 26 °C with 18 h photoperiod. One plant per pot was maintained. *Bipolaris maydis and Curvularia lunata* conidial suspension was prepared by scraping all conidia from the culture plate after 10 days of incubation followed by suspension in deionized water containing 0.02% tween 20 (Himedia, Mumbai, India). The final pathogen concentration was adjusted to 1 × 10^6^ conidia L^−1^, as suggested in earlier reports [48]. The final spore suspension concentration (10^6^ spores/mL) was inoculated on the leaves of 25-day-old maize plants using a hand atomizer until leaf run-off. Uninoculated plants were sprayed with sterile distilled water in the same way. Each treatment had three replicates, and the maize plants were kept in a glass house at 26 ± 2 °C with 12 h of darkness and 12 h of light, with the treated plants covered with polythene bags to maintain a relative humidity of more than 90% as per the method described by Manzar et al. [49]. After the infection appeared on the leaves, the fungus was re-isolated from the infected leaves and the identity was reconfirmed, to prove Koch’s postulates. The pathogenicity test was carried out thrice (Supplementary Figure S5a,b). The cultures of the isolate were deposited to the IDA-approved National Agriculturally Important Microbial Culture Collection (NAIMCC) and the accession number was obtained.

### 2.3. Molecular Characterization

#### 2.3.1. DNA Extraction, PCR, and Sequencing

Via genomic DNA extraction, the isolates were cultured on PDA medium for 15 days at 28 °C for DNA isolation. Mycelial scrapings (50–60 mg) were obtained from the fully grown cultures in the Petri plate. Harvested mycelium was transferred into an autoclaved mortar and then powdered in a pestle mortar with liquid nitrogen. To extract genomic DNA, Nucleopore GDNA Fungus Kit- NP-7006D (Genetix Biotech Asia Pvt. Ltd., New Delhi, India) was used, as described in a previous report [29]. Quantification and assessment of the quality of genomic DNA were checked using a spectrophotometer (Shimadzu, Tokyo, Japan) by recording the absorbance at 245 nm.

#### 2.3.2. Amplification and Purification of PCR Products

The isolates were amplified using the PCR primers and conditions listed in Manamgoda et al. [29], for ITS (ITS1 and ITS4), GAPDH (gpd1 and gpd2), LSU (LSU(F) and LSU^®^), and D1 and D2 region of LSU (NL1 and NL4), i.e., ITS1 (5′-TCCGTAGGTGAACCTGCGG-3′) and ITS4 (5′-TCCTCCGCTTATTGATATGC-3′), LSU(F) (5′-ACCCGCTGAACTTAAGC-3′); LSU(R) (5′-TCCTGAGGGAAACTTCG-3′); gpd1 (5′-ATACACTGCCACCCAGAAGG-3′) and gpd2 (5′-TCGATGCGAACAGTCAAGTC-3′); and NL1 (5′-GCATATCAATAAGCGGAGGAAAA-3′) and NL4 (5′-GGTCCGTGTTTCAAGACGG-3′) [29,38,50,51,52]. PCR (Peqlab-95-06002-PEQSTAR-96 gradient thermocycler, VWR International GmbH, Darmstadt, Germany) was performed in a 25 μL volume containing 1X Taq Buffer with KCl (2.5 μL), 200 µM dNTP (0.5 μL), 1.5 mM MgCl_2_ (1.5 μL), 10 pM forward primer (1 μL), 10 pM reverse primer (1 μL), 1U/reaction Taq polymerase (0.2 μL) and 1X nuclease-free water (17.3 μL) (Genetix Biotech Asia Pvt. Ltd., New Delhi, India), and 100 ng genomic DNA (1 μL). PCR conditions listed in Manamgoda et al. (2012) for ITS, GAPDH, LSU, and D1 and D2 region of LSU gene. Amplified products (10 μL) were separated from 1.5% agarose gel containing ethidium bromide (0.5 μg/mL) by gel electrophoresis performed at 2 V cm^−1^ of the gel length until the bands were resolved and visualized, documented, and photographed in the gel-documentation unit (Bio-Rad, Philadelphia, PA, USA). Gel extraction kit (Sure extract spin PCR clean/Gel extraction kit, Genetix Biotech Asia Pvt. Ltd., New Delhi, India) was used to purify ITS, LSU, GAPDH, D1 and D2 regions of LSU of the amplified PCR products. All amplicons were bidirectional-sequenced through Sanger sequencing method (Eurofins Pvt. Ltd. Bangalore, India) in order to get maximum length.

#### 2.3.3. Sequence Alignment and Phylogenetic Analyses

Newly generated ITS, GAPDH, and LSU sequences were analyzed separately with all available ex-type available sequences downloaded from GenBank and DDBJ to determine preliminary identifications of all the isolates. Raw sequences were assembled with Sequencher v. 4.9 for Windows (Gene Codes Corp., Ann Arbor, MI, USA). The assembled consensus sequences were initially aligned with MAFFT v. 7 using default settings (http://mafft.cbrc.jp/alignment/server/; accessed on 26 June 2021) and adjusted manually where necessary [53]; poorly aligned regions of the alignments and hyper-variable regions where alignment was ambiguous were removed using Trimal [54] and exported in a standard PHYLIP file. To fully resolve closely related species, all isolates were subjected to a multi-gene combined analysis. Phylogenetic reconstructions of concatenated and individual gene trees were performed using maximum likelihood (ML) criteria. Maximum likelihood trees were generated using RAxML 8.1.15 [55] under a GTRGAMMA model. Bipartitioned information was also input into RAxML software [56] for maximum likelihood (ML) analysis. A rapid bootstrap (BS) analysis was conducted with 1000 replications, the software produced a best-scoring ML tree with BS probabilities, and the results were obtained in ‘TRE’ files. The combined multi-marker dataset (ITS, GAPDH and LSU) was bipartitioned by gene region and was analyzed using RAxML as described above. Phylogenetic trees were viewed in MEGA v. 11 [57]. All sequences generated were deposited in GenBank (Table 2).

#### 2.3.4. Statistical Analysis

Statistical analysis of length and width of each isolate were analyzed by using a one-way analysis of variance [58] and Duncan’s multiple range test (DMRT) using Statistical Product and Service Solution (SPSS) version 16.0 software Developed by SPSS Inc., now IBM SPSS Armonk, NY, USA. On average, 30 measurements of conidial length and width of each isolate were recorded. Mean ± standard deviation for length and width ranges of each conidium was recorded.

**Table 2:** Details of the isolates used in this study, including the hosts, locations, and GenBank accession numbers of the generated sequences of the ribosomal marker (ITS, LSU, and D1 and D2 region of LSU) and protein-coding gene GAPDH.

Isolates Code	Host	Location	Identified Organism	ITS1 and ITS4 Accession Number	GAPDH Accession Number	LSU Accession Number	D1 and D2 Region of LSU Accession Number	NAIMCC Accession Number	Reference
E43	*Zea mays*	Hariharpur, Sant Kabir Nagar, Uttar Pradesh, India	*Curvularia lunata*	MH104636	LC543643	MT416023	LC546663	NAIMCC-F-03913	This study
E36	*Zea mays*	Ludhiana, Punjab, India	*Curvularia lunata*	MH104637	LC543644	MT416024	LC546664	Yet to be submitted	This study
E50	*Zea mays*	Pantnagar, Udham Singh Nagar, Uttarakhand, India	*Bipolaris maydis*	MH145411	LC543968	MT416015	LC546657	NAIMCC-F-03997	This study
E7	*Zea mays*	Nagla, Udham Singh Nagar, Uttarakhand, India	*Bipolaris maydis*	MH145412	LC508971	MT416016	LC546658	NAIMCC-F-03919	This study
E10	*Zea mays*	HardasChak, Khagaria, Bihar, India	*Bipolaris maydis*	MH145413	LC543969	MT416017	LC546659	NAIMCC-F-03990	This study
E15	*Zea mays*	Godargama, Begusarai, Bihar, India	*Bipolaris maydis*	MH145414	LC538220	MT416018	LC546660	NAIMCC-F-03991	This study
E13	*Zea mays*	Khargajepur, MauNath Bhanjan Uttar Pradesh, India	*Curvularia lunata*	MH183189	LC524133	MT416004	LC546646	NAIMCC-F-03917	This study
E27	*Zea mays*	KhiriyaMau Nath BhanjanUttar Pradesh, India	*Bipolaris maydis*	MH183190	LC544122	MT416005	LC546647	NAIMCC-F-03916	This study
E25	*Zea mays*	Kasuet, MaunathBhanjanUttar Pradesh, India	*Bipolaris maydis*	MH183191	LC529408	MT416006	LC546648	NAIMCC-F-03988	This study
E37	*Zea mays*	Ludhiana, Punjab, India	*Curvularia lunata*	MH183192	LC544123	MT416007	LC546649	NAIMCC-F-03915	This study
E41	*Zea mays*	Azamgarh, Uttar Pradesh, India	*Curvularia lunata*	MH183193	LC529749	MT416008	LC546650	NAIMCC-F-03914	This study
E16	*Zea mays*	Sari Tole SanicharaAsthan, Samastipur, Bihar, India	*Curvularia lunata*	MH183194	LC538355	MT416009	LC546651	Yet to be submitted	This study
E39	*Zea mays*	Mau Nath BhanjanUttar Pradesh, India	*Curvularia lunata*	MH183195	LC544124	MT416010	LC546652	Yet to be submitted	This study
E12	*Zea mays*	Singhaw, FatehpurUttarPradesh, India	*Curvularia lunata*	MH183196	LC508972	MT416011	LC546653	NAIMCC-F-03918	This study
E19	*Zea mays*	Saidpur Ama, Begusarai, Bihar	*Bipolaris maydis*	MH183197	LC541581	MT416012	LC546654	NAIMCC-F-03992	This study
E34	*Zea mays*	Ludhiana, Punjab, India	*Curvularia lunata*	MH183198	LC545392	MT416013	LC546655	Yet to be submitted	This study
E3	*Zea mays*	Godargama, Begusarai, Bihar, India	*Bipolaris maydis*	MT799977	LC538353	MT799989	LC546665	NAIMCC-F-03989	This study
E14	*Zea mays*	Lakho, Begusarai, Bihar, India	*Curvularia lunata*	MT799978	LC538354	MT799990	LC546666	NAIMCC-F-04001	This study
E24	*Zea mays*	Jigni, Rohtas, Bihar, India	*Bipolaris maydis*	MT524321	LC552295	MT516300	MT533838	NAIMCC-F-03993	This study
E30	*Zea mays*	BhindKund, Ballia, Uttar Pradesh, India	*Bipolaris maydis*	MT524322	LC552288	MT516301	MT533839	NAIMCC-F-03998	This study
E35	*Zea mays*	Ludhiana, Punjab, India	*Curvularia lunata*	MT524323	LC552292	MT516302	MT533840	Yet to be submitted	This study
E28	*Zea mays*	Bhar, MauNathBhanjan, Uttar Pradesh, India	*Curvularia lunata*	MT524324	LC552287	MT516303	MT533841	Yet to be submitted	This study
E33	*Zea mays*	Khojpur, Jalandhar, Punjab, India	*Curvularia lunata*	MT524325	LC552290	MT516304	MT533842	NAIMCC-F-03986	This study
E6	*Zea mays*	Talwandi Bharo, Jalandhar, Punjab, India	*Curvularia lunata*	MT524326	LC552294	MT516305	MT533843	Yet to be submitted	This study
E38	*Zea mays*	Jalandhar, Punjab, India	*Bipolaris maydis*	MT524327	LC552293	MT516306	MT533844	NAIMCC-F-03996	This study
E32	*Zea mays*	Allowal, Punjab, India	*Curvularia lunata*	MT524328	LC552289	MT516307	MT533845	Yet to be submitted	This study
E31	*Zea mays*	BarharaChargaha, Maharajganj, Uttar Pradesh, India	*Curvularia lunata*	MT524329	LC552683	MT516308	MT533846	NAIMCC-F-04002	This study
E26	*Zea mays*	Mustafabad, FaizabadUttar Pradesh, India	*Bipolaris maydis*	MT524331	LC552286	MT516310	MT533848	NAIMCC-F-03994	This study
AR 5183	*Zea mays*	Japan	*Bipolaris maydis*	KM230390	KM034848	NA	NA	NA	[11]
CBS 136.29	*Zea mays*	Japan	*Bipolaris maydis*	KJ909769	KM034845	NA	NA	NA	[11]
AR 5182	*Zea mays*	Japan	*Bipolaris maydis*	KM230388	KM034844	NA	NA	NA	[11]
CBS 137271/C5	*Zea mays*	USA	*Bipolaris maydis*	AF071325	KM034846	NA	NA	NA	[11]
CBS137271/C5	*Zea mays*	USA	*Bipolaris maydis*	AF071325	KM034846	KM243280	NA	NA	[11]
	*Zea mays*							NA	
CBS157.34		Indonesia	*Bipolaris maydis*	JX256430	JX276442	-	NA	NA	[42]
CPC 28832	Triticum aestivum	Thailand	*Bipolaris sorokiniana*	MF490812	MF490834	-	NA	NA	[59]
M 1122/C4	*Zea mays*	USA	*Bipolaris maydis*	KM230389	KM034847	-	NA	NA	[11]
CBS 108941	NA	=	*Dreschlera erythrospila*	AY004782	AY004813	-	NA	NA	[60]
MFLUCC 10-0706	Oryza sativa,	Thailand	*Curvularia lunata*	JX256431	JX276443	JX256398	NA	NA	[11]
CBS730.96	Human lung biopsy	USA	*Curvularia lunata*	JX256429	JX276441	JX256396	NA	NA	[11]
MFLUCC 10-0695	*Panicum* sp.	Thailand	*Curvularia lunata*	JX256432	JX276444	JX256399	NA	NA	[11]
CBS 136.29	*Zea mays*	Japan	*Bipolaris maydis*	KJ909769	KM034845	-	NA	NA	[11]
CBS 307.64	*Zea mays*	USA	*Bipolaris maydis*	HF934925	HG779085	HF934875	NA	NA	[61]
CBS 130.26	*Zea mays*	-	*Bipolaris maydis*	HF934923	HG779084	HF934873	NA	NA	[61]
CBS573.73	*Zea mays*	USA	*Bipolaris maydis*	HF934924	NA	MH872501	HF934881	NA	[61]
CBS 307.84	*Avena sativa*	Sweden	*Pyrenophora avenicola*	MK539972	MK540180	MK540042	NA	NA	[62]
CBS330.53	=	Japan	*Rhizopus oryzae*	MH857229	NA	MH868766	NA	NA	[63]
ICMP 6128	*Cynodon dactylon*	New Zealand	*Bipolaris cynodontis*	JX256412	JX276427	JX256380	NA	NA	[42]
BRIP 12898	*Melinis munitiflora*	Australia	*Bipolaris melinidis*	JN601035	JN600972	JX256411	NA	NA	[42]
CBS 280.91	*Microlaenae stipoidis*	Australia	*Bipolaris microlaenae*	JN601032	JN600974	JN600995	NA	NA	[42]
MFLUCC 10-0694	*Oryza sativa*	Thailand	*Bipolaris oryzae*	JX256413	JX276428	JX256381	NA	NA	[42]
BRIP 12790	*Cynodon dactylon*	Australia	*Bipolaris peregianensis*	JN601034	JN600977	JN601000	NA	NA	[42]
MFLUCC 10-0705	*Panicum* sp.	Thailand	*Curvularia alcornii*	JX256421	JX276434	JX256388	NA	NA	[42]
MFLUCC 10-0687	*Oryza sativa*	Thailand	*Curvularia asianensis*	JX256422	JX276435	JX256389	NA	NA	[42]
CBS 193.62	*Air*	Pakistan	*Curvularia ellisii*	JN192375	JN600963	JN600985	NA	NA	[42]
ICMP 6160	*Gladiolus* sp.	New Zealand	*Curvularia gladioli*	JX256426	JX276438	JX256393	NA	NA	[42]
BRIP 23186a	*-*	Australia	*Curvularia graminicola*	JN192376	JN600964	JN600986	NA	NA	[42]
BRIP 15933	*Chloris gayana*	Australia	*Curvularia hawaiiensis*	JN601028	JN600965	JN600987	NA	NA	[42]
CBS 284.91	*Heteropogon contortus*	Australia	*Curvularia heteropogonis*	JN192379	JN600969	JN600990	NA	NA	[42]
ICMP 6172	*Ischaemum indicum*	Solomon Islands	*Curvularia ischaemi*	JX256428	JX276440	JX256395	NA	NA	[42]
BRIP 15882a	*Eragrostis interrupta*	Australia	*Curvularia ovariicola*	JN601031	JN600971	JN600992	NA	NA	[42]
BRIP 13165a	*Sporobolus fertilis*	Australia	*Curvularia ravenelii*	JN192386	JN600978	JN601001	NA	NA	[42]
CBS 274.52a	*Soil*	Spain	*Curvularia spicifera*	JN192387	JN600979	JX256400	NA	NA	[42]
BRIP 12375	*Unknown*	Australia	*Curvularia tripogonis*	JN192388	JN600980	JN601002	NA	NA	[42]
CBS 146.63	*Unknown*	India	*Curvularia tuberculata*	MH858243	LT715830	MH869845	NA	NA	[42]
CBS 192.29		Japan	*Curvularia coicis*	MH855040	HG779130	MH866505	NA	NA	[63]
BRIP 14845	*Coffea arabica*	Kenya	*B. coffeana*	KJ415525	KJ415421	KJ415478	NA	NA	[64]
BRIP 12530	*Dactyloctenium radulan*	Australia	*B. clavate*	KJ415524	KJ415422	KJ415477	NA	NA	[64]
CBS274.91	*Eleusine indica*	USA	*Bipolaris eleusines*	KJ909768	KM034820	KM243289	NA	NA	[11]
BRIP 14838	*Croton* sp.	-	*Bipolaris crotonis*	KJ415526	KJ415420	KJ415479	NA	NA	[64]
CBS 109894	*C. dactylon*	Hungary	*Bipolaris cynodontis*	KJ909767	KM034838	KM243288	NA	NA	[11]
CBS241.92	*Hevea* sp.	Nigeria	*B.heveae*	KJ909763	KM034843	KM243294	NA	NA	[11]
BRIP 14840	*Gossypium* sp.	Kenya	*B. gossypina*	KJ415528	KJ415418	KJ415481	NA	NA	[11]
BRIP 15613	*Microlaena stipoides*	Australia	*B. microlaenae*	JN601032	JN600974	JN600995	NA	NA	[18]
CBS 199.29	*P. miliaceum*	Japan	*B. panici-miliace*	KJ909773	KM042896	KM243281	NA	NA	[18]
BRIP 12790	*C. dactylon*	Australia	*B. peregianensis*	JN601034	JN600977	JN601000	NA	NA	[18]
BRIP 14839	*E. coracana*	Zambia	*B. pluriseptata*	KJ41553	KJ415414	KJ41548	NA	NA	[64]
IMI 228224	*Salvinia auriculata*	Brazil	*B. salviniae*	KJ922390	KM034829	KM243283	NA	NA	[11]
CBS 120.24		Italy	*B. sorokiniana*	KJ909776	KM034821	KM243278	NA	NA	[11]
CBS 624.68	*Dichanthium annulatum*	USA	*C. robusta*	KJ909783	KM083613	KM243297	NA	NA	[11]
CBS 349.90	*S. creber*	Australia	*C. riley*	KJ909766	KM083612	KM243267	NA	NA	[11]
CBS 239.48	*-*	USA	*Curvularia portulacae*	MH856324	LT715903	MH867878	NA	NA	[63]
CBS 350.90a	*Perotis rara*	Australia	*Curvularia perotidis*	JN192385	HG779138	JN600999	NA	NA	[18]
CBS 156.35	*Air*	Jawa	*C. pallescens*	KJ922380	KM083606	KM243269	NA	NA	[11]
CBS 160.58	*Eleusine Indica*	USA	*C. nodulosa*	JN601033	JN600975	JN600997	NA	NA	[11]
CBS 656.74	*Desert soil*	Egypt	*C. subpapendorfii*	KJ909777	KM061791	KM243266	NA	NA	[11]
CBS 327.64	*Avena sativa*	USA	*B. victoriae*	KJ909778	KM034811	KM243271	NA	NA	[11]
BRIP 17186	*Heliconia psittacorum*	Australia	*Bipolaris heliconiae*	KJ415530	KJ415417	KJ415483	NA	NA	[64]
BRIP 15900	*Sorghum bicolor*	Australia	*Curvularia sorghina*	KJ415558	KJ415388	KJ415512	NA	NA	[64]
BRIP 14845	*Coffea arabica*	Kenya	*Bipolaris coffeana*	KJ415525	KJ415421	KJ415478	NA	NA	[64]
E29	*Zea mays*	India	*Curvularia geniculata*	MT524330	LC552684	MT533847	NA	NA	[49]

CBS: CBS-KNAW Fungal Biodiversity Centre, Utrecht, The Netherlands; MFLUCC: Mae Fah Luang University Culture Collection, Center of Excellence in Fungal Research, Thailand; BRIP: Biosecurity Queensland Plant Pathology Herbarium, Brisbane, Queensland, Australia. NA: not available.

## 3. Results

### 3.1. Morphological Identification of Bipolaris Isolates (Maydis Leaf Blight)

Twenty sampling district sites were chosen from major maize-growing regions of India, from which a total of 186 leaf blight symptomatic samples were collected. Seventy-seven isolates of *Bipolaris* were identified from the total collected maydis-leaf-blight-symptomatic samples (Table 1). The 77 isolates were divided into five groups as per cultural and morphological characteristics (Table 3). Group A consisted of 20 isolates, representing 25.97%, Group B consisted of 9 isolates, representing 11.68%, Group C consisted of 25 isolates accounting for 32.46%, Group D had 11 isolates accounting for 14.28%, and Group E consisted of 12 isolates which accounted for 15.58%. Table 3, give a brief overview of the morphological data for these *Bipolaris* isolates in groups A–E. Characteristics of the colony: variable colony morphology on PDA was found after 15 days for each group. Group A isolates had gray colonies with spots, raised mycelia was observed with irregular margin; in Group B isolates, colony color was gray with smooth raised mycelia and irregular margins. The isolates in Group C produced gray colonies with appressed mycelia and irregular margin. The colonies from Group D were blackish gray with white spot, smooth appressed mycelia, and regular margin, whereas Group E had whitish gray colonies, rough raised mycelia, and irregular margin. The conidia length-width ratio, color and shape, and conidial morphology was grouped into five groups. Group A had a larger length–width ratio than Group C and Group D. All the isolates of the five groups produced slightly curved, light-brown to brown, fusiform conidia (Supplementary Figure S2).

SEM observations: *Bipolaris* conidia had smooth walls, were fusiform or slightly curved, and placed on a geniculate conidiophore. *Bipolaris* spp. conidial attachment to conidiophores, conidial hilum, and conidia were all seen (Supplementary Figure S3).

### 3.2. Morphological Identification of Curvularia Isolates (Maize Leaf Spot)

A total of 129 maize-leaf-spot-symptomatic samples were collected from 20 sampling districts from five major maize-growing regions and 74 *Curvularia* isolates were identified based on their morphological characteristics (Table 4). Seventy-four isolates were divided into five groups based on their cultural and morphological characteristics. Group F consisted of 15 isolates, representing 20.27%, Group G included 8 isolates, accounting for 10.81%, Group H consisted of 21 isolates, accounting for 28.37%, Group I had 14 isolates, accounting for 18.91%, and Group J consisted of 16 isolates, which accounted for 21.62%. A brief description of the cultural and morphological characteristics of *Curvularia* isolates as described in F–J groups can be found in Table 4. Different colony morphology was found on PDA for each distinct group after 7 days of incubation. Group F isolates had black colonies and smooth velvety mycelia with regular margin; Group G isolates had colonies that were gray in color with smooth floccose mycelia and irregular margins. The isolates in Group H produced gray colonies with smooth appressed mycelia and irregular margin. The colonies from Group I were blackish gray with smooth appressed mycelia and regular margin. The colonies produced by Group J isolates were whitish gray with smooth velvety mycelia and regular margin. Conidial morphology: conidial types were observed among the five groups based on the following conidia shapes, color and length–width ratio: The length–width ratio of Group I was larger than those of Group G and Group J. All Group isolates produced 3–5 distoseptate conidia with light-brown to brown color (Supplementary Figure S4).

### 3.3. Pathogenicity of the Isolate

Koch’s postulates experiments were performed for the isolates and were positive for causing MLB disease and leaf spot of maize. The maydis leaf blight symptoms appeared as small yellow necrotic spots that later become spindle- or elliptical-shaped lesions and were observed in the inoculated plants, *Zea mays* c.v. Kanchan (Supplementary Figure S5a). In maize leaf spot, the symptoms appeared as yellow necrotic spots in the early stages of maize leaves, which eventually expanded to circular, oval, or strip lesions formed after 10 days of inoculation, identical to those seen in the field for leaf spot of maize. (Supplementary Figure S5b).

### 3.4. Phylogenetic Study of Bipolaris Isolates (Causal Organism of Maydis Leaf Blight)

PCR-amplified product of *Bipolaris* spp. and *Curvularia* spp. using primer pairs ITS1/ITS4, GAPDH1/GAPDH2, D1/D2 LSU yielded specific amplified products of approximately 650 bp (ITS), 472 bp (*gapdh*), and 917 bp (LSU). Maximum likelihood analyses were performed both individually and in combination based on the collected sequenced data to produce phylograms. Forty-three ex-type closely related species of *Bipolaris* and *Curvularia* were used as reference sequences. The isolates were characterized at molecular level using thee three above-mentioned gene loci; upon NCBI BLAST search, they showed 99–100% similarity with concern pathogen and were identified as *Bipolaris maydis* and *Curvularia lunata*. These isolates were submitted to GenBank and IDA (International Depositary Authority)-approved culture collection center NAIMCC after approval of cultures authenticity from the authority. Accession no. of culture collection is mentioned and described in Table 2.

#### 3.4.1. Phylogeny Based on the Ribosomal Marker ITS Regions of *Bipolaris maydis*

**Figure 1:**
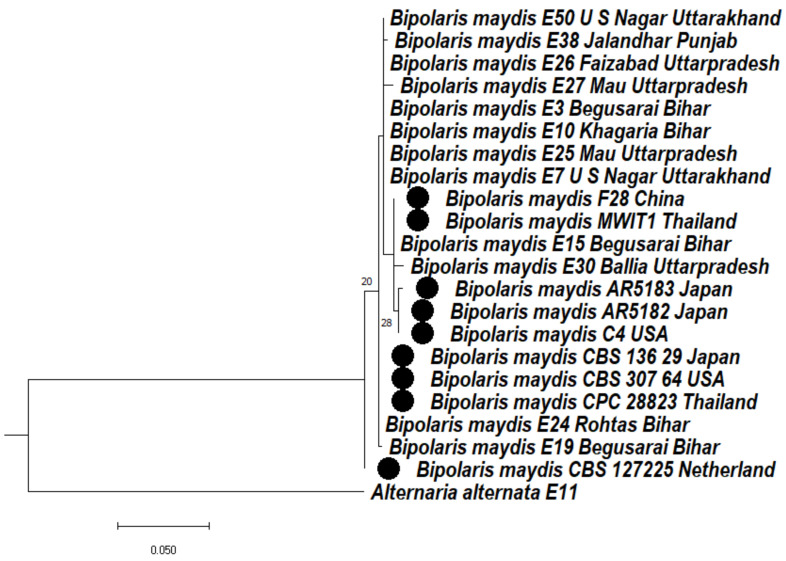
Dendrogram constructed from maximum likelihood method based on combined internal transcribed spacer (ITS). Sequenced data of the *Bipolaris maydis* isolates were inferred with Raxml based on GTR + Gamma model. Numerical value presented in the node indicates bootstrap value with a 1000 non-parametric bootstrap replicate analysis. The symbol ● denotes as ‘*ex-type*’ closely related species sequences obtained from GenBank. *Alternaria alternata* used as an outgroup.

Based on the sequences of the ITS regions of *Bipolaris* isolates, a phylogenetic tree was made to ascertain the relationship among the isolates of *Bipolaris maydis*. In the phylogenetic tree, two clades were formed. In clade 1, two subclades were formed; subclade 1A formed two subclades, 1Aa and 1Ab. In subclade 1Aa, *Bipolaris maydis* isolates E50, E38, E26, E27, E3, E10, E25, E7 were clustered together, whereas F28, MWT1, E15, E30 were clustered together, AR5183, AR5182, C4 were clustered together, and CBS136.29, CBS-307.64, CPC-28823, E24 were clustered together. Subclade 1Ab consists of E19. Clade 1B consists of CBS127225. *Alternaria alternata* (E11) was used as an outgroup in clade 2 (Figure 1).

#### 3.4.2. Phylogenetic Analysis of ITS + GAPDH Gene of *Bipolaris maydis*

**Figure 2:**
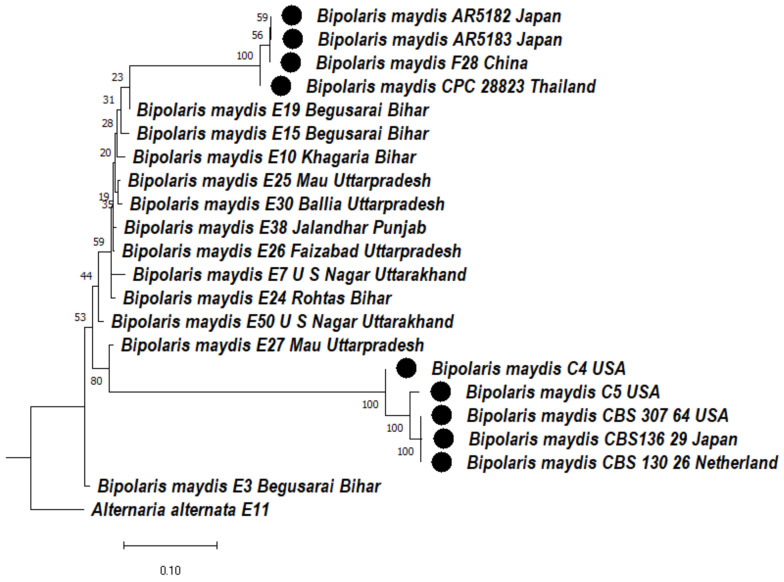
Dendrogram constructed from maximum likelihood method based on combined internal transcribed spacer (ITS) and glyceraldehyde-3-phosphate dehydrogenase (GAPDH). Sequenced data of the *Bipolaris maydis* isolates were inferred with Raxml based on GTR + Gamma model. Numerical value presented in the node indicates bootstrap value with a 1000 non-parametric bootstrap replicate analysis. The symbol ● denotes as ‘*ex-type*’ closely related species sequences obtained from GenBank. *Alternaria alternata* used as an outgroup.

A phylogenetic tree was constructed based on the aligned sequences of ITS + GAPDH region to ascertain the phylogenetic relationship between the *Bipolaris maydis* isolates. The cladogram was divided into two clades. In clade 1, there are two subclades: in subclade 1Aa, the *Bipolaris maydis* isolates AR5182, AR5183, F28 were clustered together with a bootstrap value of 100% and CPC28823, E19, E15, E10, E25, E30, E38, E26, E7, E24 formed a sister clade with them, whereas subclade 1Ab consists of E50 isolate, which formed a distinct clade. Subclade 1Ba consists of E27 isolate with a bootstrap value of 80%, subclade 1Bb consists of CBS307.64, CBS136.29, CBS130.26, which were clustered together with a bootstrap value of 100%, whereas C5, C4 formed a sister clade with them, with a bootstrap value of 100%. Subclade 2 consists of E3 isolate. *Alternaria alternata* E11 was used as an outgroup in clade 2 (Figure 2).

#### 3.4.3. Phylogeny Based on the ITS + LSU Gene of *Bipolaris maydis*

**Figure 3:**
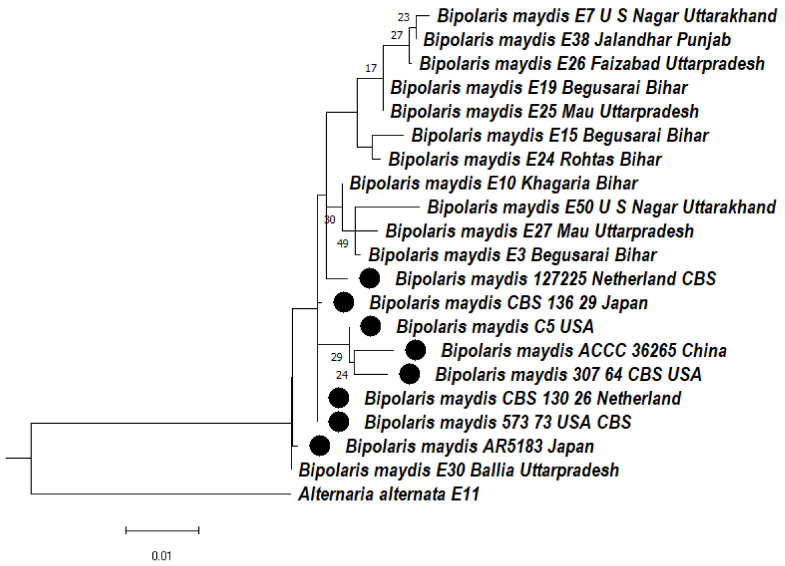
Dendrogram constructed from maximum likelihood method based on combined internal transcribed spacer (ITS) and large Subunit gene regions (LSU). Sequenced data of the *Bipolaris maydis* isolates were inferred with Raxml based on GTR + Gamma model. Numerical value presented in the node indicates bootstrap value with a 1000 non-parametric bootstrap replicate analysis. The symbol ● denotes as ‘*ex-type*’ closely related species sequences obtained from GenBank. *Alternaria alternata* used as an outgroup.

The phylogenetic tree was constructed based on the aligned sequences of ITS + LSU region to ascertain the evolutionary relationship among the *Bipolaris maydis* isolates. The cladogram was divided into two clades. Clade 1 divided into two subclades (1 and 2). In subclade 1, two subclades were formed, 1A and 1B. In subclade 1Aaa, *Bipolaris maydis* isolates E7, E38, E26 were clustered together, whereas E19, E25 formed a sister clade with them, and E15, E24 were clustered together. In subclade 1Abb, E50, E27, E3 were clustered together, whereas E10 formed a distinct clade with them. Subclade 1Ab consists of 127, 225 isolates. Subclade 1B consists of CBS 136.29, whereas in subclade 1C, C5, ACCC36265, 307.64 were clustered together. In subclade 1D, *Bipolaris maydis* isolates CBS130.26, CBS 573.73, AR 5183 formed a distinct clade. Subclade 2 consists of E30 isolate. In clade 2, *Alternaria alternata* (E11) was used as an outgroup that formed a separate clade (Figure 3).

#### 3.4.4. Phylogeny Based on the ITS + LSU + GAPDH Gene of *Bipolaris maydis*

**Figure 4:**
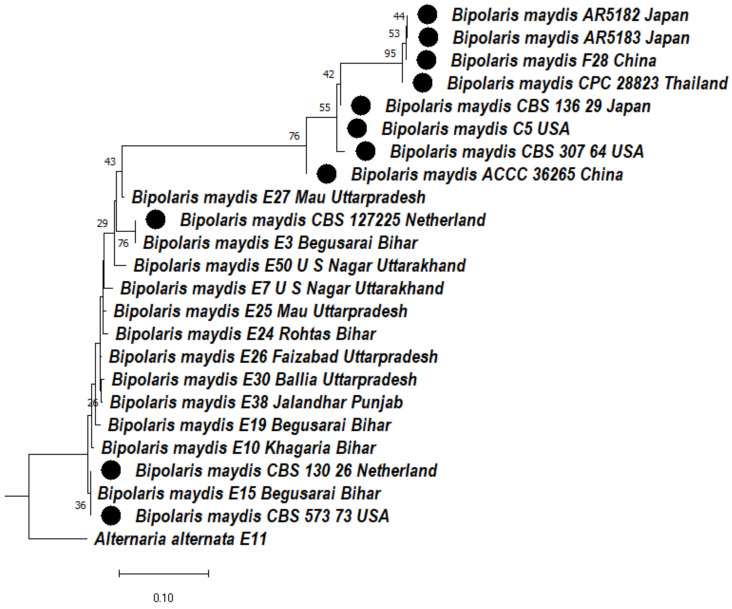
Dendrogram constructed from maximum likelihood method based on combined internal transcribed spacer (ITS) and large Subunit gene regions (LSU) and glyceraldehyde-3-phosphate dehydrogenase (GAPDH). Sequenced data of the *Bipolaris maydis* isolates were inferred with Raxml based on GTR + Gamma model. Numerical value presented in the node indicates bootstrap value with a 1000 non-parametric bootstrap replicate analysis. The symbol ● denotes as ‘*ex-type*’ closely related species sequences obtained from GenBank. *Alternaria alternata* used as an outgroup.

A cladogram was constructed based on the concatenated aligned multilocus sequences of *Bipolaris maydis* isolates to ascertain the phylogenetic relationship among the *Bipolaris maydis* isolates. The cladogram was divided into two clades (Figure 4). In clade 1, two subclades formed. Subclade 1A consists of subclade 1Aa and 1Ab; in subclade 1Aa, AR5182, AR518312, F28, CPC28823 were clustered together with a bootstrap value of 95% and CBS136.29, CBS307.64, C5 were clustered together, whereas in subclade 1Ab, ACCC36265, E27 formed a sister clade with them with a bootstrap value of 76%. In subclade 1B, *Bipolaris maydis* isolates CBS127225, E3 were clustered together with a bootstrap value of 76%, whereas E50, E7, E25, E24, E26, E30, E38, E19, E10 formed a sister clade with them. In subclade 1C, CBS130.26, E15, CBS573.73 were clustered together. In clade 2, E11 *Alternaria alternata* was used as an outgroup that formed a separate clade (Figure 4).

### 3.5. Phylogenetic Study of Curvularia lunata Isolates (Causal Organism of Maize Leaf Spot)

#### 3.5.1. Phylogeny Based on the ITS of *Curvularia lunata*

**Figure 5:**
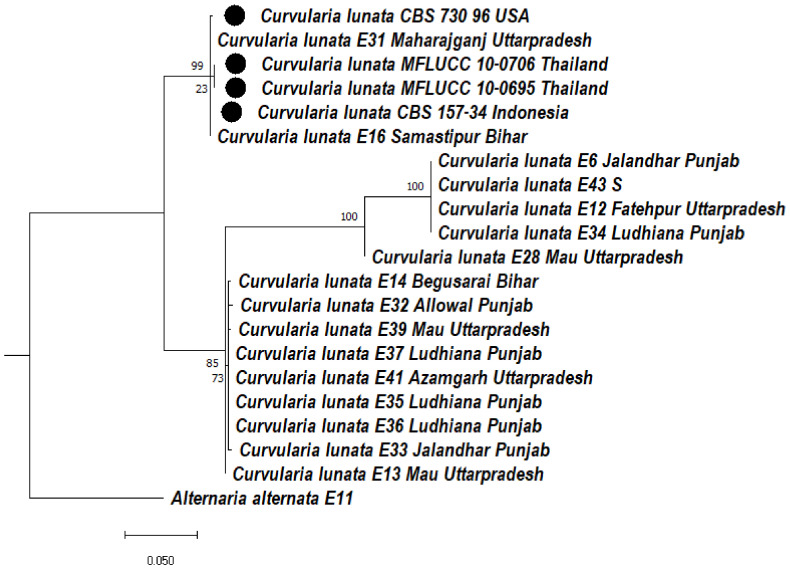
Dendrogram constructed from maximum likelihood method based on combined internal transcribed spacer (ITS), and large subunit gene regions (LSU) and glyceraldehyde-3-phosphate dehydrogenase (GAPDH). Sequenced data of the *Curvularia lunata* isolates were inferred with Raxml based on GTR + Gamma model. Numerical value presented in the node indicates bootstrap value with a 1000 non-parametric bootstrap replicate analysis. The symbol ● denotes as ‘*ex-type*’ closely related species sequences obtained from GenBank. *Alternaria alternata* used as an outgroup.

Based on the ITS region, the *Curvularia lunata* (maize leaf spot) isolates formed two clades. In clade 1, two subclades, 1A and 1B, were formed. In subclade 1A, two subclades, 1A and 1B, were formed. In subclades 1A, *Curvularia lunata* isolates CBS730.96, E31, CBS 157-34, E16 were clustered together, whereas MFLUCC 10-0706, MFLUCC 10-0695 were clustered together with a bootstrap value of 99%. In subclade 1Ba, E6, E43, E12, E34 were clustered with each other and formed a distinct clade with a bootstrap value of 100%, whereas E28 formed a distinct clade with a bootstrap value 100%. In subclade 1Bb, E14, E32, E39, E37, E41, E35, E36, E33 isolates formed a distinct clade with a bootstrap value 85%, whereas E13 formed a distinct clade with a bootstrap value of 73%. In clade 2, E11 *Alternaria alternata* was used as an outgroup that formed a separate clade (Figure 5).

#### 3.5.2. Phylogeny Based on the ITS + GAPDH of *Curvularia lunata*

**Figure 6:**
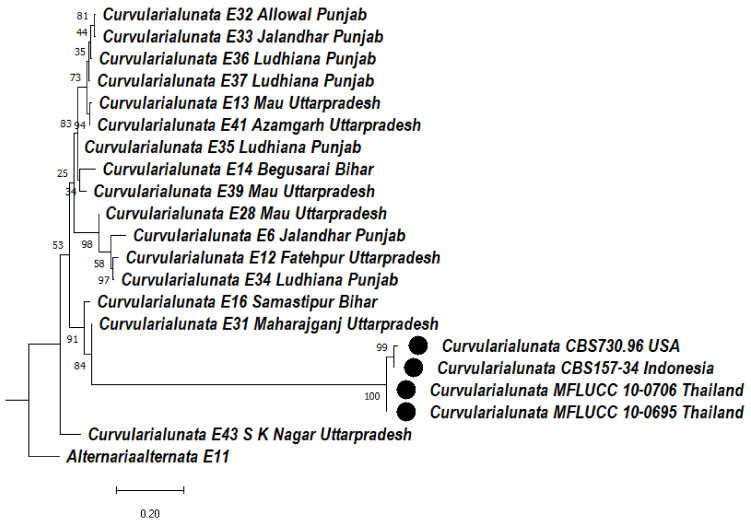
Dendrogram constructed from maximum likelihood method based on combined internal transcribed spacer (ITS) and glyceraldehyde-3-phosphate dehydrogenase (GAPDH). Sequenced data of the *Curvularia lunata* isolates were inferred with Raxml based on GTR + Gamma model. Numerical value presented in the node indicates bootstrap value with a 1000 non-parametric bootstrap replicate analysis. The symbol ● denotes as ‘*ex-type*’ closely related species sequences obtained from GenBank. *Alternaria alternata* used as an outgroup.

Based on the ITS + GAPDH region, the *Curvularia lunata* isolates formed two clades formed. In clade 1, there are two subclades, 1 and 2. In subclade 1A, E32, E33 isolates were clustered together with a bootstrap value of 81%, whereas E36, E37 formed a sister clade with them with a bootstrap value of 73%; E13, E41 were clustered together with a bootstrap value of 94% and formed a sister clade with them. In subclade 1B, E35, E14, E39, were clustered together. In subclade 1C, E28, E6, E12, E34 were clustered together with a bootstrap value of 98%. Subclade 1Da consists of E16 with a bootstrap value of 91% and subclade 1Db consists of E31 with a bootstrap value of 84%, whereas in subclade 1Dc, CBS 730.96, CBS 157-34 were clustered together with a bootstrap value of 99% and MFLUCC 10-0706, MFLUCC 10-0695 were clustered together with a bootstrap value of 100%. Subclade 2 consists of *Curvularia lunata* isolate E43. In clade 2, *Alternaria alternata* (E11) was used as an outgroup that formed a separate clade (Figure 6).

#### 3.5.3. Phylogeny Based on the ITS + LSU of *Curvularia lunata*

**Figure 7:**
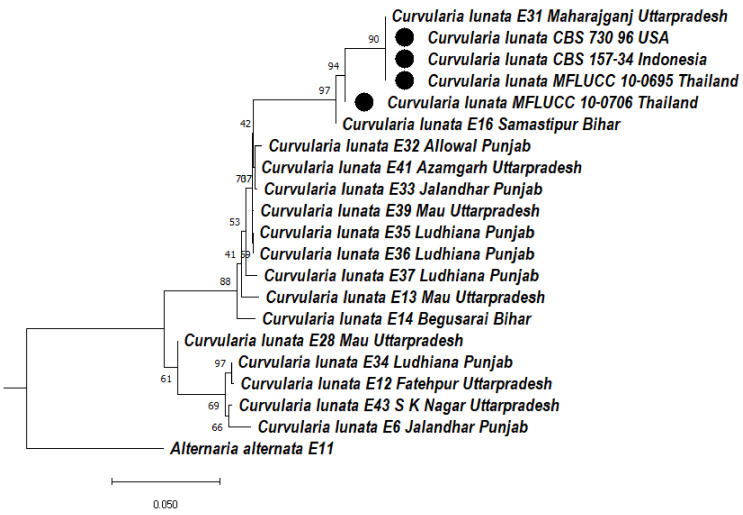
Dendrogram constructed from maximum likelihood method based on combined internal transcribed spacer (ITS) and large subunit gene regions (LSU). Sequenced data of the *Curvularia lunata* isolates were inferred with Raxml based on GTR + Gamma model. Numerical value presented in the node indicates bootstrap value with a 1000 non-parametric bootstrap replicates analysis. The symbol ● denotes as ‘*ex-type*’ closely related species sequences obtained from GenBank. *Alternaria alternata* used as an outgroup.

Based on the ITS + LSU region, two clades were formed; in clade 1, two subclades formed, subclade 1 and subclade 2. In subclade 1Aa, *Curvularia lunata* isolates E31, CBS 730.96, CBS 157-34, MFLUCC 10-0695 were clustered together with a bootstrap value of 90%, MFLUCC 10-0706 formed a sister clade with a bootstrap value of 94%, and E16 formed a sister clade with a bootstrap value of 97%. In subclade 1Ab, E32, E41, E33 were clustered together with a bootstrap value of 76% and E39, E35, E36 formed a sister clade with them, whereas E37, E13, E14 formed a distinct clade with a bootstrap value of 88%. Subclade 1Ca consists of E28 with a bootstrap value of 61%, whereas in subclade 1Cb, E34, E12 were clustered together with a bootstrap value of 97% and E43, E6 were clustered together with a bootstrap value of 69%. In clade 2, *Alternaria alternata* (E11) was used as an outgroup to form a distinct clade (Figure 7).

#### 3.5.4. Phylogeny Based on the Multigene of *Curvularia lunata*

**Figure 8:**
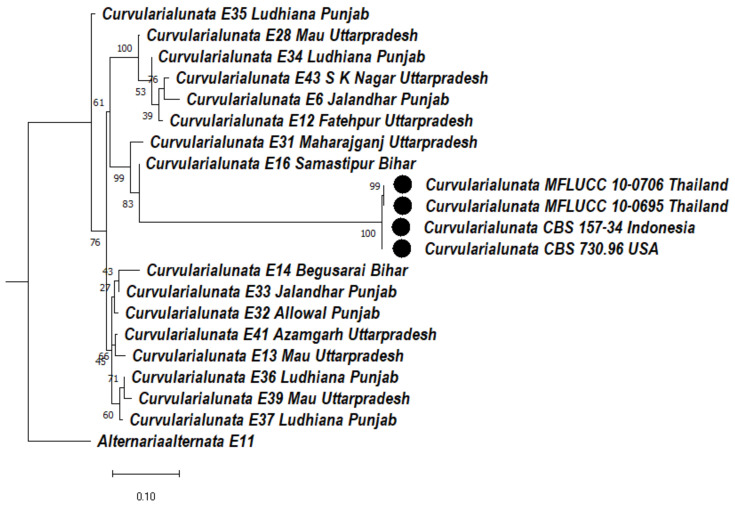
Dendrogram constructed from maximum likelihood method based on combined internal transcribed spacer (ITS), large subunit gene regions (LSU), and glyceraldehyde-3-phosphate dehydrogenase (GAPDH). Sequenced data of the *Curvularia lunata* isolates were inferred with Raxml based on GTR + Gamma model. Numerical value presented in the node indicates bootstrap value with a 1000 non-parametric bootstrap replicate analysis. The symbol ● denotes as ‘*ex-type*’ closely related species sequences obtained from GenBank. *Alternaria alternata* used as an outgroup.

A cladogram was constructed based on the concatenated sequences of ITS, LSU, and GAPDH gene regions. Two clades were formed, clade 1 and clade 2. In clade 1 there are two subclades, subclade 1 and subclade 2. In subclade 1, *Curvularia lunata* isolate E35 formed a distinct clade. In subclade 2Aa, E28 formed a distinct clade with a bootstrap value of 100%. In subclade 2Ab, E34 formed a distinct clade, E43, E6 were clustered together with a bootstrap value of 76%, and E12 formed a sister clade with them. In subclade 2Aca, two clades formed. In subclade 2Ac, E31 formed a clade with a bootstrap value of 99%; in subclade 2Acb, MFLUCC 10-706, MFLUCC 10-0695 were clustered together with a bootstrap value of 99% and CBS 157-34, CBS 730.96 were clustered with each other and formed a distinct clade with a bootstrap value of 96%, whereas E16 formed a sister clade with them with a bootstrap value of 83%. In subclade 2Ad, E14, E33 were clustered together and E32 formed a sister clade with them, whereas E41, E13 were clustered together with a bootstrap value of 66%, and E36, E39 were clustered together with a bootstrap value of 71% and E37 formed a sister clade with them. In clade 2, *Alternaria alternata* (E11) was used as an outgroup formed a distinct clade (Figure 8).

### 3.6. Phylogenetic Studies between Bipolaris and Curvularia Complex

Based on the combined sequences of ITS gene regions of *Bipolaris maydis* and *Curvularia lunata*, a cladogram was constructed based on the multilocus sequences to know the phylogenetic relationship among the 26 isolates of *Bipolaris maydis* and 21 isolates of *Curvularia lunata. Rhizopus oryzae* was used as an outgroup, and 43 ex-type closely related *Curvularia* and *Bipolaris* species were used as a reference sequence.

#### 3.6.1. Phylogeny Based on the Combined Sequences of ITS + GAPDH of *Bipolaris maydis* and *Curvularia lunata*

A multilocus phylogenetic analysis of the combined sequences of the ITS and GAPDH gene regions was performed among isolates of *Bipolaris maydis* and *Curvularia lunata*. *Drechslera* species were used as a related genera and *Rhizopus oryzae* was used as an outgroup to determine the phylogenetic relationship among the isolates of *Bipolaris maydis* and *Curvularia lunata*. On the basis of the phylogenetic tree, two separate clades formed. In clade 1, two subclades formed, subclade 1 and subclade 2. Subclade 1A consists of *Drechslera erythospila* isolate with a bootstrap value of 66%, subclade 1Ba consist of distinct genus *Curvularia lunata* isolates E43, E6, E13, E41, E37, E36, E32, E33, E39, E14, E35, which were clustered together with a bootstrap value of 91%, whereas subclade 1Bb formed with a genus of *Bipolaris maydis* isolates E27, E3, E50, E25, E10, E30, E15, E19, E26, E38, E24, E7, with *Bipolaris eleusines* CBS274.91 clustered together. In subclade 1Bc, *Curvularia lunata* isolates E16, E31, E28, E12, E34 were clustered together with a bootstrap value of 100%.

Subclade 2A consists of *Curvularia subpapendorfii* (CBS 656.74), *Curvularia spicifera* (CBS 274.52), *Curvularia sorghina* (BRIP15900), *Curvularia lunata* (CBS 730.96) *Curvularia lunata ex-type* species MFLUCC 10-0695, MFLUCC 10-0706, CBS 157-34, MFLUCC 10-0706, CBS157-34, *Curvularia ischaemi* (ICMP 6172), *Curvularia gladioli* (ICMP 6160), *Curvularia geniculata* (E29), *Curvularia pallescens* (CBS 156.35), *Curvularia heteropogonis* (CBS 284.91), *Curvularia graminicola* (BRIP 23186a), *Curvularia portulacae* (CBS 239.48). In subclade 2B, *Bipolaris maydis* isolates CBS 307.64, C5, C4, *Bipolaris oryzae* (MFLUCC 10-0694), *Bipolaris panici-miliacei* (CBS 199.29), *Bipolaris cynodontis* (CBS 109894), *Bipolaris cynodontis* (ICMP 6128), *Bipolaris sorokiniana* (CBS 120.24), *Bipolaris victoriae* (CBS 327.64), *Bipolaris maydis* (CBS 136.29), *Bipolaris crotonis* (BRIP 14838), *Bipolaris gossypina* (BRIP 14840), *Bipolaris heliconiae* (BRIP 17186), *Bipolaris clavata_*(BRIP 12530), *Bipolaris coffeana* (BRIP 14845), *Bipolaris pluriseptata* (BRIP 14839), *Bipolaris melinidis* (BRIP12898), *Bipolaris peregianensis* (BRIP 12790), *Bipolaris microlaenae* (CBS 280.91), *Bipolaris microlaenae* (BRIP 15613) were clustered together, whereas *Drechslera avenae* (CBS279.31) formed a sister clade with them with a bootstrap value of 97%. Subclade 2C consists of *Curvularia tuberculata* (CBS 146.63) and *Curvularia nodulosa* (CBS 160.58), which were clustered together with a bootstrap value of 88%; *Curvularia ovariicola*_(BRIP 15882), *Curvularia tripogonis* (BRIP 12375), *Curvularia ravenelii* (BRIP 13165), *Curvularia ryleyi* (CBS 349.90), *Curvularia asianensis* (MFLUCC 10-0687), *Curvularia coicis* (CBS 192.29), *Curvularia robusta* (CBS 624.68), *Curvularia hawaiiensis* (BRIP 15933), *Curvularia perotidis* (CBS 350.90), *Curvularia ellisii* (CBS 193.62) were clustered together.

**Figure 9:**
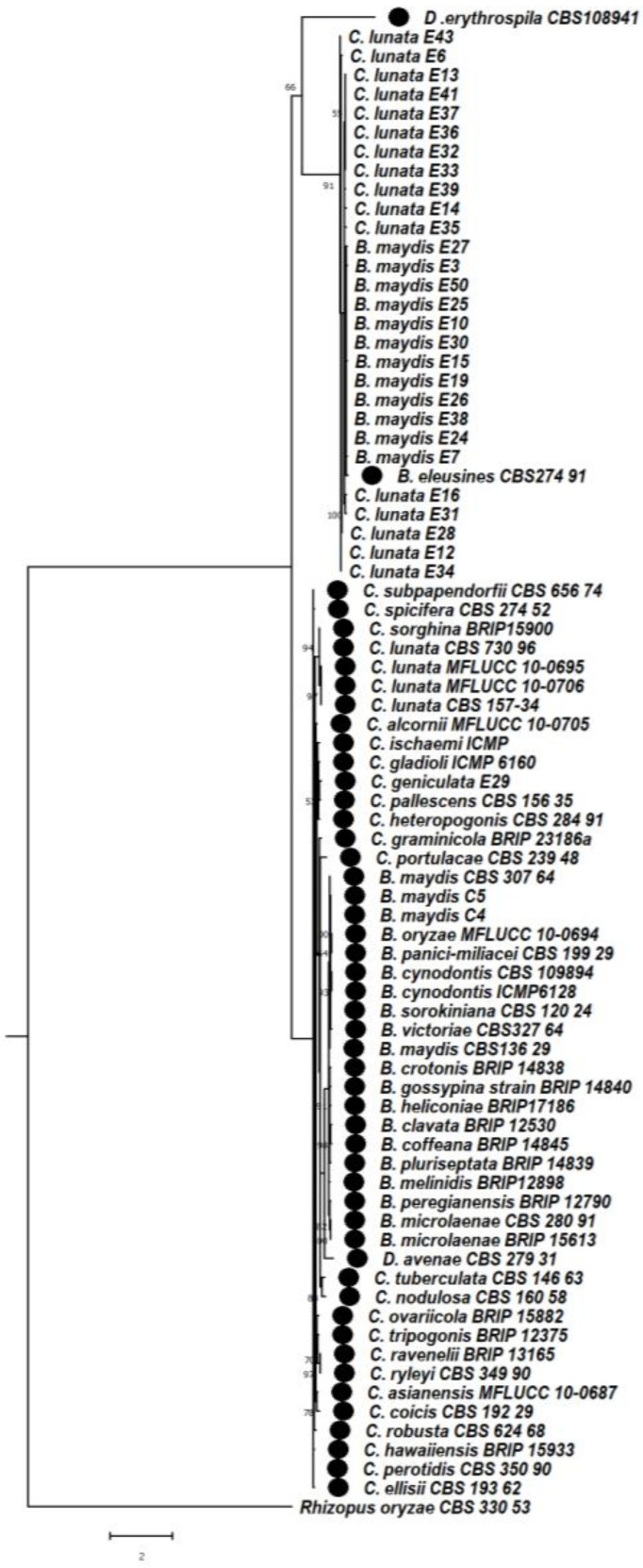
Maximum likelihood phylogeny based on combined internal transcribed spacer (ITS) and (GAPDH). Sequenced data of the *Bipolaris maydis* and *Curvularia lunata* isolates were inferred with Raxml based on GTR + Gamma model. Numerical value presented in the node indicates bootstrap value with a 1000 non-parametric bootstrap replicates analysis. *Drechslera avenae* CBS 279.31, *Drechslera erythospila* (sexual stage-*Pyrenophora erythospila)* CBS 108,941 were used as other genera from Helminthosporoid group. The symbol ● denotes as ‘*ex-type*’ 43 closely related species sequences obtained from GenBank. *Rhizopus oryzae* used as an outgroup.

In clade2, *Rhizopus oryzae* (CBS 330.53) is considered as an outgroup which is entirely different from Helminthosporoid genera (Figure 9).

#### 3.6.2. Phylogeny Based on the Combined Sequences of ITS + LSU of *Bipolaris maydis* and *Curvularia lunata*

**Figure 10:**
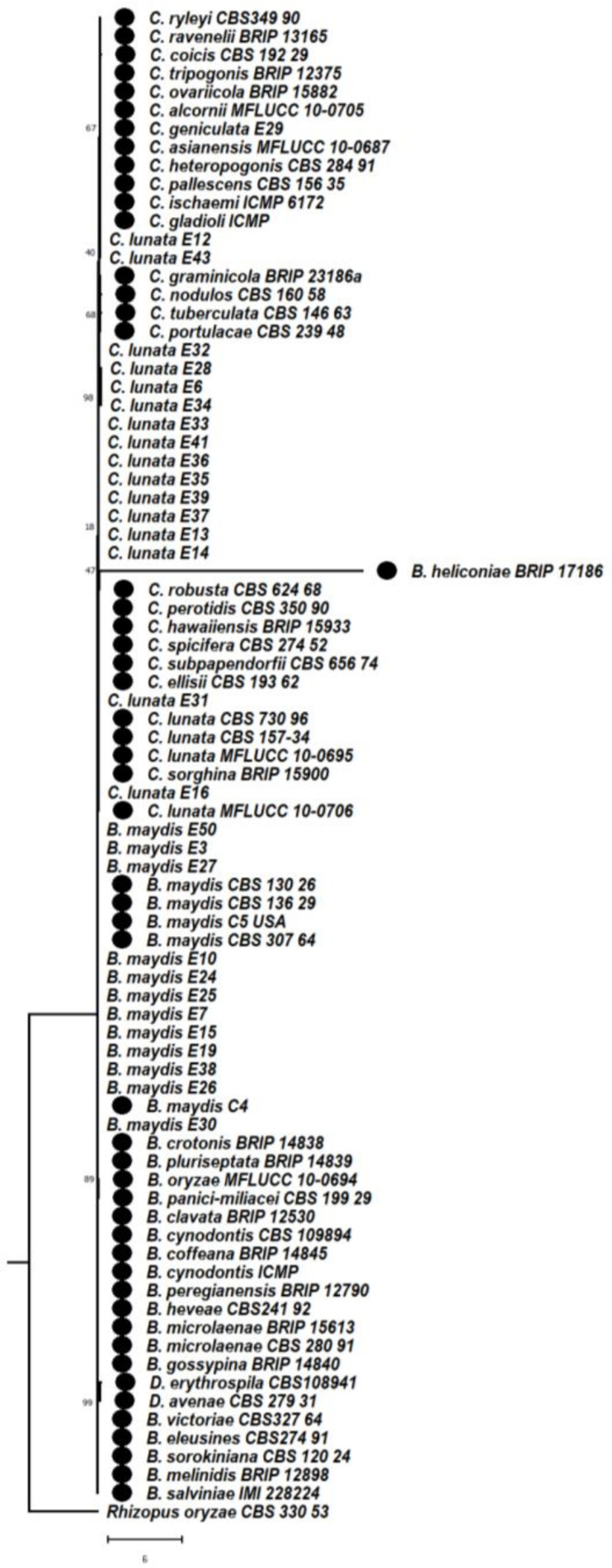
Maximum likelihood phylogeny based on combined internal transcribed spacer (ITS) and large subunit gene regions (LSU). Sequenced data of the *Bipolaris maydis*, and *Curvularia lunata isolates* were inferred with Raxml based on GTR + Gamma model. Numerical value presented in the node indicates bootstrap value with a 1000 non-parametric bootstrap replicate analysis. *Drechslera avenae* CBS 279.31, *Drechslera erythospila* (sexual stage-*Pyrenophora erythospila)* CBS 108,941 were used as other genera from Helminthosporoid group. The symbol ● denotes as ‘*ex-type*’ 43 closely related species sequences obtained from GenBank. *Rhizopus oryzae* used as an outgroup. *Rhizopus oryzae* used as an outgroup.

A multilocus phylogenetic analysis of the combined sequences of the ITS and LSU gene regions was performed on the basis of the phylogenetic tree, and two clades were formed. Clade 1A consists of *Curvularia ryleyi* (CBS 349.90), *Curvularia ravenelii* (BRIP 13165), *Curvularia coicis* (CBS192.29), *Curvularia tripogonis* (BRIP12375), *Curvularia ovaricola* (BRIP15882), *Curvularia alcornii* (MFLUCC 10-0705), *Curvularia geniculata* (E29), *Curvularia asianensis* (MFLUCC 10-0687), *Curvularia pallescens* CBS 156.35, *Curvularia heteropogonis* (CBS 284.91), *Curvularia ischaemi* (ICMP 6172), *Curvularia gladioli* (ICMP 6160), *Curvularia lunata* isolates E12, E43, *Curvularia graminicola*_(BRIP 23186a), *Curvularia nodulosa* (CBS 160.58), *Curvularia tuberculata* (CBS 146.63), *Curvularia portulacae* (CBS 239.48), *Curvularia lunata* isolates E32, E28, E6, E34, E33, E41, E36, E35, E39, E37, E13, E14, *Bipolaris heliconiae* (BRIP 17186), *Curvularia robusta* (CBS 624.68), *Curvularia perotidis* (CBS 350.90), *Curvularia hawaiiensis* (BRIP 15933), *Curvularia spicifera* (CBS 274.52), *Curvularia ellisii* (CBS 193.62), *Curvularia lunata* isolates:E31, CBS 730.96, CBS 157.34, MFLUCC 10-0695, MFLUCC 10-0706, E16, *Curvularia subpapendorfii* (CBS 656.74), *Curvularia sorghina* (BRIP 15900), *Bipolaris maydis* isolates E50, E3, E27, CBS 136.29, CBS 130.26, C5, CBS307.64, E10, E24, E25, E7, E15, E19, E38, E26, C4, E30, *Bipolaris crotonis* (BRIP 14838), *Bipolaris pluriseptata* (BRIP 14839), *Bipolaris oryzae* (MFLUCC 10-0694), *Bipolaris panici-miliacei* (CBS 199.29), *Bipolaris clavata*_(BRIP 12530), *Bipolaris cynodontis* (ICMP 6128), *Bipolaris coffeana* (BRIP 14845), *Bipolaris cynodontis* (CBS 109894), *Bipolaris peregianensis* (BRIP 12790), *Bipolaris heveae* (CBS241.9), *Bipolaris microlaenae* (CBS 280.91), *Bipolaris microlaenae* (BRIP 15613), *Bipolaris gossypina* (BRIP 14840), *Drechslera avenae* (CBS 279.31), *Drechslera erythospila* (sexual stage-*Pyrenophora erythospila* CBS108941), *Bipolaris victoriae*_(CBS327.64), *Bipolaris melinidis* (BRIP 12898), *Bipolaris sorokiniana* (CBS 120.24), *Bipolaris salviniae* (IMI 228224), which were clustered with each other. In clade 2, *Rhizopus oryzae* (CBS 330.53) is considered as an outgroup which is entirely different from Helminthosporoid genera (Figure 10).

#### 3.6.3. Phylogeny Based on the Combined Regions Multigene for Bipolaris and Curvularia Complex

A multilocus phylogenetic analysis of the concatenated sequences of ITS + GAPDH + LSU region was performed among the *Bipolaris maydis* and *Curvularia lunata* isolates, where *Drechslera avenae* and *Rhizopus oryzae* were used as an outgroup. On the basis of the phylogenetic tree, two separate clades formed. In clade 1, three subclades were formed; in clade 2, *Rhizopus oryzae* (CBS 330.53) was considered as an outgroup which is completely different from Helminthosporoid genera.

In subclade 1Aa, *Curvularialunata* (MFLUCC10-0695), *Curvularialunata* (MFLUCC10-0706), *Curvularia lunata* isolates (CBS730.96), (CBS157.34) were clustered together. In subclade1Ab, *Bipolaris maydis* isolates CBS 307.64, CBS 136.29, C5, CBS 157.34, CBS 130.26, *Bipolaris sorokiniana* (CBS 120.24), *Bipolaris eleusines* (CBS274.91), *Bipolaris pluriseptata* (BRIP 14839), *Bipolaris clavata* (BRIP 12530), *Bipolaris coffeana* (BRIP 14845), *Bipolaris panici-miliacei* (CBS 199.29), *Bipolaris oryzae* (MFLUCC 10-0694), *Bipolaris heveae* (CBS 241.92), *Bipolaris heliconiae* (BRIP 17186), *Bipolaris gossypina* (BRIP 14840), *Bipolaris crotonis* (BRIP 14838), *Bipolaris microlaenae* (CBS 280.91), *Bipolaris microlaenae* (BRIP 15613), *Bipolaris peregianensis* (BRIP 12790), *Bipolaris melinidis* (BRIP 12898), *Bipolaris salviniae* (IMI 228224), *Bipolaris eleusines* (CBS274.91), *Bipolaris pluriseptata* (BRIP 14839), *Bipolaris clavata* (BRIP 12530), *Bipolaris coffeana* (BRIP 14845), *Bipolaris cynodontis* (ICMP 6128), *Bipolaris cynodontis* (CBS 109894) were clustered together. In subclade 1Ac, *Curvularia tuberculata* (CBS 146-63), *Curvularia graminicola* (BRIP 23186a), *Curvularia nodulosa* (CBS 160.58), *Curvularia portulacae* (CBS 239.48), *Curvularia geniculata* (E29), *Drechslera avenae* (CBS 279.31), *Drechslera erythospila* (sexual stage-*Pyrenophora erythospila)* (CBS 108941), *Curvularia heteropogonis* (CBS289.91), *Curvularia ovaricola* (BRIP15882), *Curvularia tripogonis* (BRIP 12,375), *Curvularia sorghina* (BRIP15900), *Curvularia coicis* (CBS 192.29), *Curvularia ischaemi* (ICMP 6172), *Curvularia asianensis* (MFLUCC 10-0687), *Curvularia alcornii* (MFLUCC 10-0705) *Curvularia gladioli* (ICMP), *Curvularia pallescens* (CBS 156.35), *Curvularia ryleyi* (CBS 349.90), *Curvularia ravenelli* (BRIP 13165) were clustered together.

In subclade 2Aa, *Curvularia lunata* isolates E34, E12, E43, E6, E28, E16, E31 were clustered together. In subclade 2Ab, *Bipolaris maydis* isolates E50, E27, E3, E24, E25, E15, E7, E10, E19, E26, E30, E38 were clustered together. In subclade 2Ac, *Curvularia lunata* isolates E35, E37, E13, E33, E39, E36, E41, E14, E32 were clustered together.

**Figure 11:**
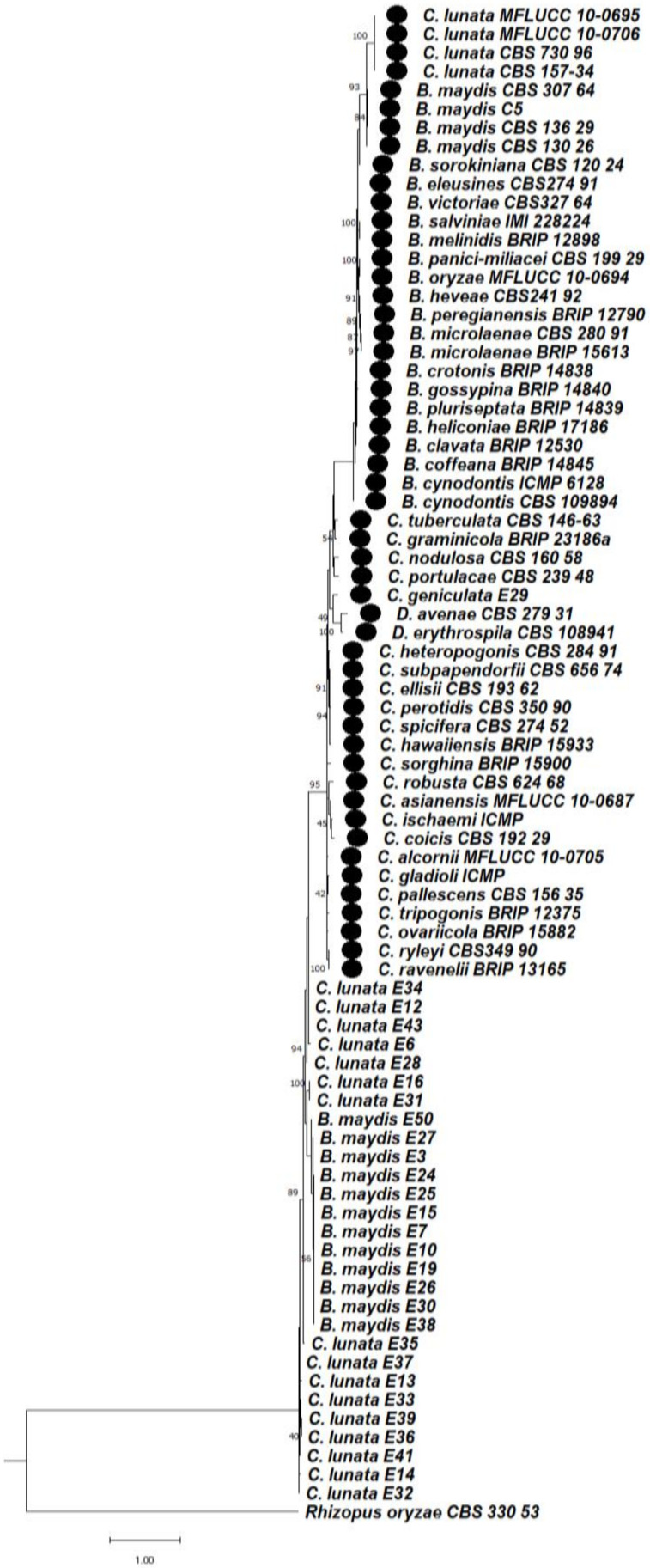
Maximum likelihood phylogeny based on combined internal transcribed spacer (ITS), large subunit gene regions (LSU), and glyceraldehyde-3-phosphate dehydrogenase (GAPDH). Sequenced data of the *Bipolaris maydis* and *Curvularia lunata isolates* were inferred with Raxml based on GTR + Gamma model. Numerical value presented in the node indicates bootstrap value with a 1000 non-parametric bootstrap replicate analysis. *Drechslera avenae* CBS 279.31, *Drechslera erythospila* (sexual stage-*Pyrenophora erythospila)* CBS 108,941 were used as other genera from Helminthosporoid group. The symbol ● denotes as ‘*ex-type*’ 43 closely related species sequences obtained from GenBank. *Rhizopus oryzae* used as an outgroup.

In clade 2, *Rhizopus oryzae* (CBS 330.53) is considered as an outgroup that is entirely different from Helminthosporoid genera (Figure 11).

## 4. Discussion

*Bipolaris* and *Curvularia* species that are anamorphs of *Cochliobolus* species are important pathogens of the Poaceae family. Leaf spots, blight, root rot, and crown rots are diseases caused by these pathogens [11,12,38]. Payak and Sharma (1981) reported 61 diseases in maize [65]. Among the diseases, maydis leaf blight causes yield losses of up to 41 percent [20]. Considerable phenotypic, pathogenic, and genetic variations occur among the pathogens under the genus *Helminthosporium* [66,67,68]. *Bipolaris maydis*, previously called *Helminthosporium maydis*, also shows enormous variability. The studies on diversity analysis based on morphological variation and phylogenetic basis in *B. maydis-Zea mays* pathosystem have not been carried out very extensively. Keeping this fact in mind, the present study was undertaken, and the results are here discussed. The present work focused on elucidating the biodiversity of the *Bipolaris maydis*, causing maydis leaf blight, and *Curvularia lunata*, causing maize leaf spot. To this end, a vast collection of *Bipolaris maydis* isolates obtained mainly from maize from the major maize-growing regions of Bihar, Punjab, Uttar Pradesh, and Uttarakhand were characterized based on morphological and molecular characteristics.

Seventy-seven maydis leaf blight isolates were morphologically characterized and formed into five groups based on their morphology. The data showed that different isolate lengths and widths of conidia varied between 65.38–80.44 μm and 12.43–14.64 μm, respectively (Table 1). In *Bipolaris maydis* isolates, Group A had the largest size of conidia, having a mean length of 80.44 μm and width of 14.64 μm, whereas in Group B, the size of conidia was the smallest (65.38 × 12.43 μm) and showed a distinct variation among the isolates based on conidia length and width. Similar observations were made by Sun et al., 2020; Pal et al., 2015; Leonard and Suggs, 1974 [14,69,70]. Comparison of the conidium sizes of the isolates of *Bipolaris maydis* and *Curvularia lunata* examined in this study was consistent with results from previous studies [12,71,72]. The number of septa of various isolates ranged between four and nine, and the average number of septa was highest for Group E (5–9); in Group D, the number of septa was smallest (4–5). The isolates showed two textural classes, namely rough and smooth, and colony pattern was either appressed or raised type. Group A and Group E isolates showed rough texture with raised colony pattern, whereas Group C showed smooth texture with an appressed colony. Group A, B, C, and E grown on PDA showed regular margins, while Group D exhibited irregular margins. Similar variations in number of septa among the isolates of *Bipolaris maydis* were found in the same agroclimatic zones [14,70,73].

In *Curvularia lunata* isolates, Group H had the largest size of conidia, having a mean length of 29.15 μm and width of 11.96 μm, whereas in Group I, the size of conidia was smallest (19.69 × 9.58 μm) and showed a distinct variation among the isolates based on conidia length and width. The distoseptate conidia were smooth-walled and curved at subterminal cell, three-septate, four-celled, and brown. The Group H, and Group I produced grey, smooth mycelia, whereas Group F produced black smooth velvety mycelia. These morphological characters are similar to the characters of *Curvularia lunata*-caused leaf spot of maize described earlier [74,75] and standard descriptions from Ellis (1971) [10].

Our study mainly highlights the correct way of identifying of *Bipolaris maydis* and *Curvularia lunata* isolates, as both show complex symptoms in field conditions that can cause confusion in their identification. In this study, we screened out indigenous isolates based on morphological characteristics and ribosomal markers ITS and LSU, while protein-coding GAPDH genes were used for molecular identification and phylogenetic analysis. A phylogenetic tree was constructed based on the concatenated aligned multigene sequences of ribosomal markers ITS and LSU and protein-coding GAPDH gene regions of *Bipolaris maydis* isolates and found that maydis leaf blight isolates of Bihar, Uttar Pradesh, and Punjab isolates were clustered together, whereas Uttatrakhand isolates were clustered only with Bihar and Uttarpradesh isolates. In other words, Upper Gangetic Plain region and Middle Gangetic Plain region isolates of *Bipolaris maydis* showed similarity with each other. Whiles *Bipolaris maydis* isolates constructed phylogeny only on the basis ITS sequences, the Bihar, Uttarpradesh, Punjab, and Uttarakhand isolates clustered together, and the aligned multigene sequences of ribosomal marker ITS and protein-coding GAPDH gene regions revealed that the isolates of *Bipolaris maydis* from other countries such as Japan, China, USA, and the Netherlands did not cluster with the Indian isolates, but when phylogeny was constructed based only on ITS sequences, *Bipolaris maydis* isolates from Japan, China, USA, and the Netherlands clustered with the Indian isolates. This indicates that nuclear ribosomal DNA (rDNA) internal transcribed spacer (ITS) sequences alone could not differentiate the isolates based on their geographical locations, but that combined ITS, LSU, and GAPDH phylogeny was able to differentiate among the isolates based on said locations. A cladogram was constructed based on the concatenated sequences of ribosomal markers ITS and LSU and protein-coding GAPDH gene regions of *Curvularia lunata* isolates. The combined analysis of ITS, LSU, and GAPDH phylogeny of *Curvularia lunata* isolates revealed that most of the isolates of Punjab and Uttarakhand were clustered together, whereas Bihar isolates only clustered with Uttarpradesh isolates. Similarly, the *Curvularia lunata* isolates of Thailand, Indonesia, and USA were clustered together but did not cluster with Indian-origin isolates. However, when phylogeny was made on the basis of ITS sequences or combined sequences of ITS and LSU the *Curvularia lunata* isolates of Thailand, Indonesia, and USA were clustered with Bihar and Uttarpradesh isolates. Similar observations were found as reported earlier [11,14,29].

The only morphological distinction between *Curvularia* and the helminthosporioid genus *Bipolaris* is the conidial curvature and length. Both genera have species with intermediate morphology, requiring sequencing data to distinguish them adequately. The overlapping morphological features of several *Curvularia* taxa make species identification difficult [20,66]. Only morphological evidence with the subjective determination of phenotypic characteristics such as spore size has been used to identify *Bipolaris* and *Curvularia* spp., while some were identified entirely on the basis of host relationship. Several duplicate sequences of *Bipolaris* spp. with different names were submitted in GenBank. When searching for generic placement, ITS BLAST search should be used because it is the most efficient. Notably, Xue et al. (2016) included eight species of *Bipolaris* in their phylogenetic analysis, which led to incorrect identification of two of the three strains. Therefore, sequencing data are critical for reliable species identification with ITS, GAPDH, and LSU gene regions [28], although the ITS and GAPDH alone can still resolve the majority of taxa in *Curvularia* [26]. Although the ITS regions alone can resolve the majority of taxa in *Curvularia* [26], sequences of GAPDH and LSU regions are needed to clarify if it is a different species. In our study, for accurate identification of *Curvularia lunata* and *Bipolaris maydis* isolates, phylogenetic analyses using multi-genes (ITS or LSU) or protein-coding genes such as GAPDH were performed; the multigene analysis highlights the similarity and relationships between different isolates from different geographical locations [26,38,43].

## 5. Conclusions

It is difficult to differentiate sister genera (*Bipoplaris* and *Curvularia*) solely based on morphological examination, because many of their morphological features overlap. Therefore, other identification methods, such as molecular identification, are necessary for identification. Here, we used GAPDH, a protein-coding gene, and other ribosomal markers like ITS or LSU for phylogenetic analysis in *Bipolaris* and *Curvularia* spp. to ensure precise identification. We used precise taxon sampling for accurate results by adding 43 ex-type closely related *Bipolaris* and *Curvularia* spp. sequences from GenBank that derived from type material to identify the delineate species in *Bipolaris* and *Curvularia*. Based on the phylogenetic tree, multigene analysis (ITS + LSU + GAPDH) yielded accurate identification of fungal isolates such as *B*. *maydis* and *C*. *lunata*. While the multigene analysis highlights the similarity and relationships between the different isolates from different geographical locations, the information is comparatively degenerate in single-gene-based phylogeny. In this study, an unclear bifurcation of *Drechslera* genera was also observed in the case of (ITS + GAPDH) phylogeny, as *Drechslera* *erythrospila* CBS108941 were clustered with *Curvularia lunata*, but this case was resolved after using a multigene phylogenetic approach (ITS + LSU + GAPDH). Upon so doing, the *Drechslera* genera formed a separate cluster, which gives a clue that single- or oligogene-based molecular identification may be incorrect sometimes for correct identification. Nonetheless, multiple-gene-based concatenated phylogenies are much accurate and useful for identification in cases such as the sister genera *Bipolaris-Curvularia* complex, and they also can be utilized to differentiate isolates of fungi from different geographical locations. This research contributes to understanding *Bipolaris maydis* and *Curvularia lunata* maize disease complexity and offers an approach for correct diagnosis using concatenated gene sequence analysis. We found that *B*. *maydis* and *C*. *lunata* were the dominant species infecting maize in all geographical locations surveyed of major maize-growing regions. Future studies should focus on expanding genetic information on *Bipolaris* and *Curvularia* spp., and proper identification should continue to rely on molecular data, not on morphological parameters. The habitat and the host range of many *Bipolaris* species are incompletely understood. Extensive sampling and accumulation of molecular data will improve understanding of the host range and ecological significance of *Bipolaris* and *Curvularia* spp.

## Figures and Tables

**Table 1:** Sampling sites of maydis leaf blight and maize leaf spot samples from major maize-growing regions of India.

S. No.	Sampling Sites	District Coordinates (Latitude and Longitude)	Maydis-Leaf-Blight-Symptomatic Samples (186)	Maize Leaf Spot Samples (129)
1.	Jaunpur, Uttar Pradesh	25.62 N; 82.98 E	09	07
2.	Sant Kabir Nagar, Uttar Pradesh	25.59 N; 82.98 E	06	04
3.	Faizabad, Uttar Pradesh	26.73 N; 82.15 E	04	03
4.	Maharajganj, Uttar Pradesh	27.22 N; 83.82 E	09	05
5.	MauNathBhanjan, Uttar Pradesh	25.87 N; 83.47 E	15	04
6.	Ballia, Uttar Pradesh	25.77 N; 84.16 E	13	07
7.	Azamgarh, Uttar Pradesh	25.73 N; 82.99 E	09	05
8.	Udham Singh Nagar, Uttarakhand	28.99 N; 79.51 E	13	05
9.	Almora, Uttarakhand	29.62 N; 79.67 E	07	08
10.	Haldwani, Uttarakhand	29.16 N; 79.51 E	06	03
11.	Samastipur, Bihar	25.87 N; 85.81 E	08	11
12.	Khagaria, Bihar	25.51 N; 86.50 E	12	07
13.	Begusarai, Bihar	25.38 N; 86.18 E	18	12
14.	Rohtas, Bihar	24.63 N; 83.92 E	06	06
15.	Baruni, Bihar	25.45 N; 86.00 E	09	07
16.	Bhagalpur, Bihar	25.25 N; 86.95 E	06	04
17.	Katihar, Bihar	25.39 N; 87.63 E	08	02
18.	Ludhiana, Punjab	30.90 N; 75.79 E	09	11
19.	Jalandhar, Punjab	30.99 N; 75.74 E	11	06
20.	Allowal, Punjab	31.15 N; 75.67 E	08	12

**Table 3:** Morphological characteristics of *Bipolaris* isolates.

Group	Colony Characteristics	Conidia		
		Average Length (µm)	Average Width (µm)	Shape
A (*n* = 20)	Grey with white spots, rough raised mycelia, irregular margin	80.44 ± 0.71 ^a^ (64–89)	14.64 ± 0.28 ^ab^ (13–16)	Fusiform, slightly curved, dark brown, 4–9 distoseptate
B (*n* = 9)	Grey, smooth raised mycelia, irregular margin	65.38 ± 0.64 ^e^ (53–71)	12.43 ± 0.59 ^d^ (11–13)	Fusiform, slightly curved, light brown, 4 distoseptate
C (*n* = 25)	Grey, smooth appressed mycelia, irregular margin	74.05 ± 0.14 ^c^ (63–97)	15.14 ± 0.35 ^a^ (14–16)	Brown, 6–8 distoseptate
D (*n* = 11)	Blackish grey with white spot, smooth appressed mycelia, regular margin	76.68 ± 0.54 ^b^ (68–84)	13.44 ± 0.44 ^c^ (12–14)	Brown, 4–5 distoseptate
E *(n* = 12)	Whitish grey, rough raised mycelia, irregular margin	71.29 ± 0.80 ^d^ (51–83)	13.89 ± 0.38 ^bc^ (12–15)	Light brown, 5–9 distoseptate

The values in the column represent the average of 30 measurements, followed by the standard deviation. Duncan’s multiple range tests (DMRT) show that values with different alphabetical (a–e) superscripts within a column are significantly different (*p* ≤ 0.05).

**Table 4:** Morphological characteristics of *Curvularia* isolates.

Group	Colony Characteristics	Conidia		
		Average Length	Average Width	Shape
F (*n* = 15)	Black, smooth velvety mycelia, regular margin	20.86 ± 0.85 ^bc^ (19–22)	10.23 ± 0.35 ^b^ (8–11)	Light brown, 3–4 distoseptate
G (*n* = 8)	Grey, smooth floccose mycelia, irregular margin	27.29 ± 0.79 ^a^ (25–29)	12.71 ± 0.72 ^a^ (12–13)	Light yellow, 3–4 distoseptate
H (*n* = 21)	Grey, smooth appressed mycelia, irregular margin	29.15 ± 0.27 ^a^ (25–35)	11.96 ± 0.78 ^a^ (10–12)	Brown, 4–5 distoseptate
I (*n* = 14)	Blackish grey, smooth appressed mycelia, regular margin	19.69 ± 0.46 ^c^ (16–24)	9.58 ± 0.44 ^b^ (9–10)	Light brown, 3–4 distoseptate
J (*n* = 16)	Whitish grey, smooth velvety mycelia, regular margin	22.82 ± 0.24 ^b^ (22–23)	8.77 ± 0.67 ^b^ (8–9)	Light brown, 3–4 distoseptate

The values in the column represent the average of 30 measurements, followed by the standard deviation. Duncan’s multiple range tests (DMRT) show that values with different alphabetical (a–c) superscripts within a column are significantly different (*p* ≤ 0.05).

## Data Availability

Not applicable.

## Supplementary Materials

The following supporting information can be downloaded at: https://www.mdpi.com/article/10.3390/jof8080802/s1, Figure S1: Sampling district location shown in Map of India.; Figure S2: Representative *Bipolaris* isolates showing the morphological description from Group A to E; Figure S3: Scanning electron microscope showing conidiophore, conidia and hilum of *Bipolaris* isolates; Figure S4: Representative isolates of *Curvularia* spp. showing the morphological descriptions from Group F- Group J; Figure S5: (5a-a) Pot experiment for pathogenicity proving of maize leaf spot (b) maize leaf spot. symptomatic leaves (c) Fully grown *Curvularia lunata* culture plate (d) Microscopic image of conidia of *Curvularia lunata* at 40X; (5b-a) Pot experiment for pathogenicity proving of maydis leaf blight (b) maydis leaf blight symptomatic leaves (c) Fully grown *Bipolaris maydis* culture plate (d) Microscopic image of conidia of *Bipolaris maydis* at 40X.
