# Supplementary material for: Multi-Gene Phylogenetic Approach for Identification and Diversity Analysis of Bipolaris maydis and Curvularia lunata Isolates Causing Foliar Blight of Zea mays
Source: J Fungi (Basel). 2022 Jul 29;8(8):802. doi: 10.3390/jof8080802 (PMC9410300; doi:10.3390/jof8080802)
Supplement: Supplementary file 1 [file jof-08-00802-s001.zip › jof-1746521-supplementary.pdf]

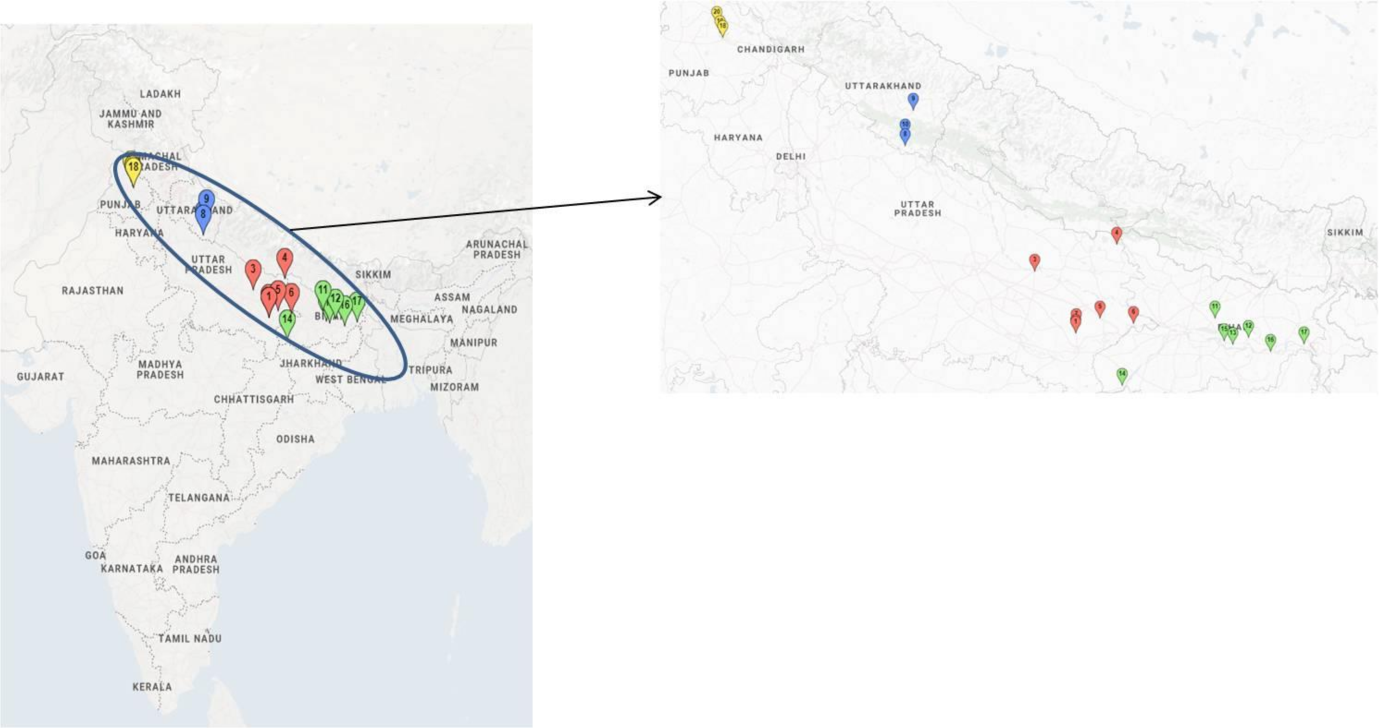

Supplementary Figure S1: Sampling district location shown in Map of India.

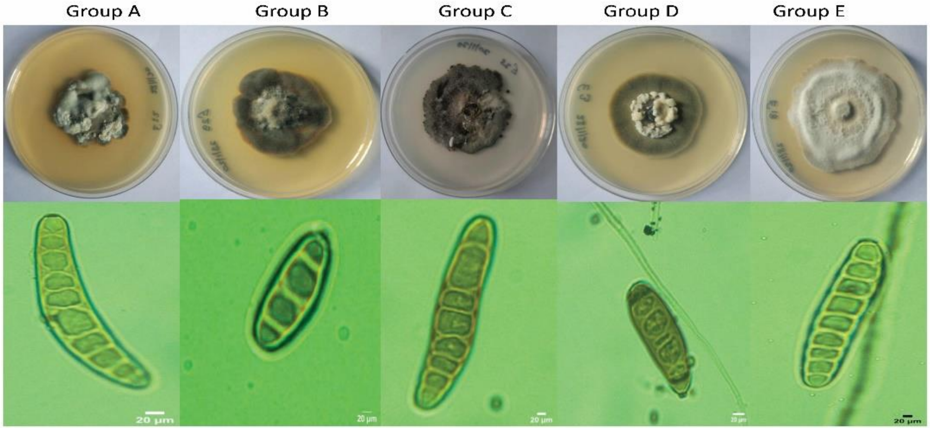

Supplementary Figure S2. Representative *Bipolaris* isolates showing the morphological description from Group A to E

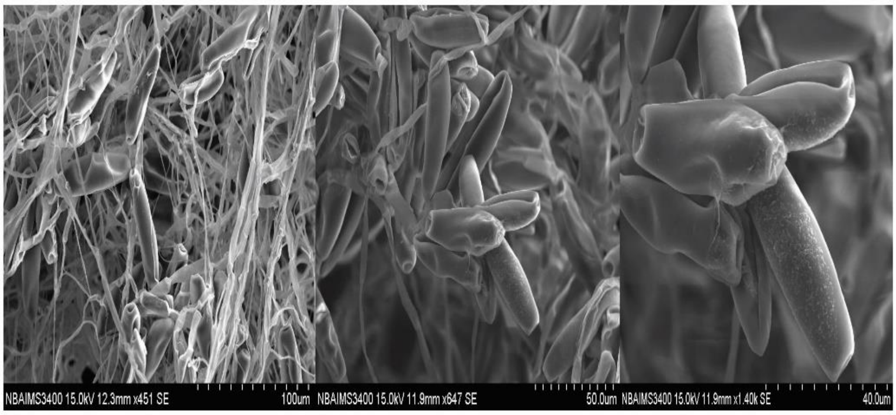

Supplementary Figure S3. Scanning electron microscope showing conidiophore, conidia and hilum of *Bipolaris* isolates.

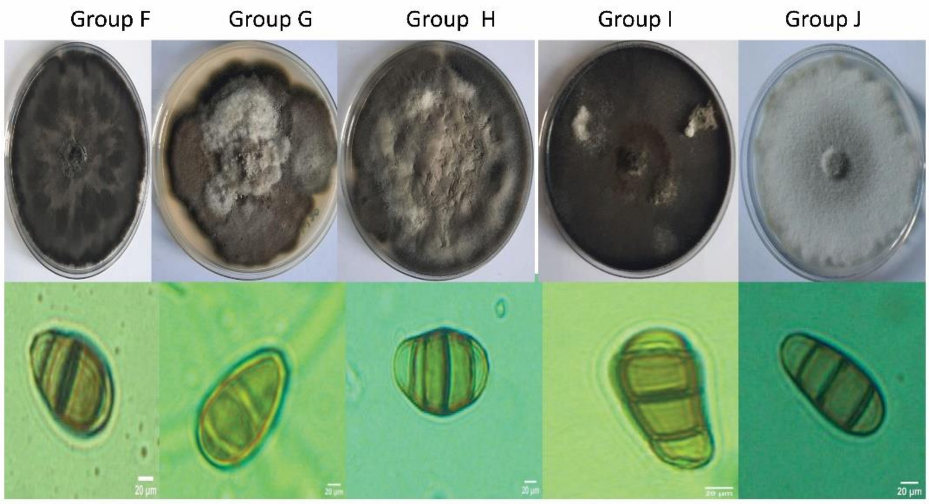

Supplementary Figure S4. Representative isolates of *Curvularia* spp. showing the morphological descriptions from Group F- Group J.

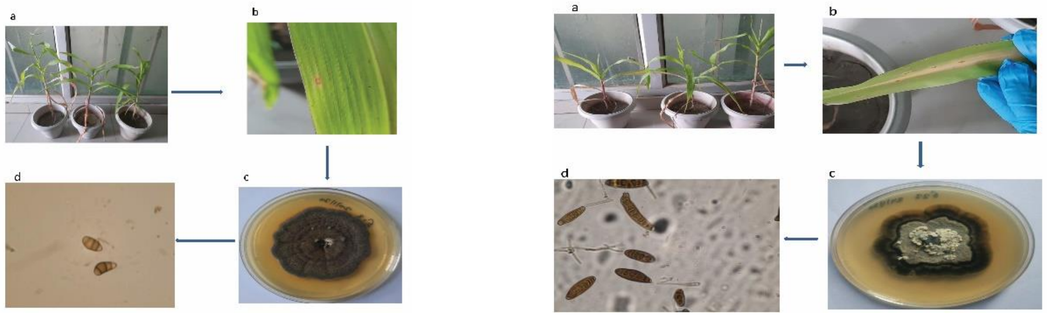

(**5a**) (**5b**)

Supplementary Figure S5. 5a-a) Pot experiment for pathogenicity proving of maize leaf spot b) maize leaf spot. symptomatic leaves c) Fully grown *Curvularia lunata* culture plate d) Microscopic image of conidia of *Curvularia lunata* at 40X; 5b-a) Pot experiment for pathogenicity proving of maydis leaf blight b) maydis leaf blight symptomatic leaves c) Fully grown *Bipolaris maydis* culture plate d)Microscopic image of conidia of *Bipolaris maydis* at 40X.
